# Molecular characterisation of formalin-fixed paraffin-embedded (FFPE) breast tumour specimens using a custom 512-gene breast cancer bead array-based platform

**DOI:** 10.1038/bjc.2011.355

**Published:** 2011-11-08

**Authors:** M Abramovitz, B G Barwick, S Willis, B Young, C Catzavelos, Z Li, M Kodani, W Tang, M Bouzyk, C S Moreno, B Leyland-Jones

**Affiliations:** 1 VM Institute of Research, 2020 University Street, Montreal, Quebec H3A 2A5, Canada; 2 Emory Biomarker Service Centre, Emory University, 1365C Clifton Road, N.E., Atlanta, GA 30322, USA; 3 Scripps Research Institute Florida, 130 Scripps Way, Jupiter, FL 33458, USA; 4 Department of Pathology, McGill University, Montreal, Quebec, Canada; 5 Department of Pathology and Laboratory Medicine, Emory University School of Medicine, Atlanta, GA 30322, USA; 6 Winship Cancer Institute, Robert W. Woodruff Health Sciences Centre, Emory University School of Medicine, 1365C Clifton Road, N.E., Atlanta, GA 30322, USA

**Keywords:** breast cancer, DASL assay, bead array, formalin-fixed, paraffin-embedded (FFPE), relapse-free survival (RFS), overall survival (OS)

## Abstract

**Background:**

Formalin-fixed, paraffin-embedded (FFPE) tumour tissue represents an immense but mainly untapped resource with respect to molecular profiling. The DASL (cDNA-mediated Annealing, Selection, extension, and Ligation) assay is a recently described, RT–PCR-based, highly multiplexed high-throughput gene expression platform developed by Illumina specifically for fragmented RNA typically obtained from FFPE specimens, which enables expression profiling. In order to extend the utility of the DASL assay for breast cancer, we have custom designed and validated a 512-gene human breast cancer panel.

**Methods:**

The RNA from FFPE breast tumour specimens were analysed using the DASL assay. Breast cancer subtype was defined from pathology immunohistochemical (IHC) staining. Differentially expressed genes between the IHC-defined subtypes were assessed by prediction analysis of microarrays (PAM) and then used in the analysis of two published data sets with clinical outcome data.

**Results:**

Gene expression signatures on our custom breast cancer panel were very reproducible between replicates (average Pearson's *R*^2^=0.962) and the 152 genes common to both the standard cancer DASL panel (Illumina) and our breast cancer DASL panel were similarly expressed for samples run on both panels (average *R*^2^=0.877). Moreover, expression of *ESR1*, *PGR* and *ERBB2* corresponded well with their respective pathology-defined IHC status. A 30-gene set indicative of IHC-defined breast cancer subtypes was found to segregate samples based on their subtype in our data sets and published data sets. Furthermore, several of these genes were significantly associated with overall survival (OS) and relapse-free survival (RFS) in these previously published data sets, indicating that they are biomarkers of the different breast cancer subtypes and the prognostic outcomes associated with these subtypes.

**Conclusion:**

We have demonstrated the ability to expression profile degraded RNA transcripts derived from FFPE tissues on the DASL platform. Importantly, we have identified a 30-biomarker gene set that can classify breast cancer into subtypes and have shown that a subset of these markers is prognostic of OS and RFS.

It is estimated that in the United States nearly 40 000 women die from breast cancer every year, making breast cancer the second most frequent cause of cancer death (behind lung) among women (Jemal *et al*, 2008). Recent publications have methodically shown the heterogeneity of this disease (Wood *et al*, 2007) and a subsequent disparity in pathological course manifested between race/ethnicities (Carey *et al*, 2006; Stead *et al*, 2009).

Currently, breast cancer is divided into major subgroups based on the combined expression of the oestrogen receptor (ER), progesterone receptor (PR) and human epidermal growth factor receptor 2 (HER2). These subgroups have important implications in breast cancer aetiology, the systemic therapies prescribed and their expected effectiveness, and in the clinical outcome measured in both recurrence-free survival (RFS) and overall survival (OS) (Sørlie *et al*, 2001, 2006). The hormone receptor-positive (HR+) (ER+ and/or PR+), HER2-negative (HER2−) subtype appears to account for the majority of breast cancers (>50%) and has the best prognosis because of effective targeted hormonal therapies and a more indolent disease phenotype. The two HER2+ subtypes (HR+/HER2+ and HR−/HER2+) account for ∼7% and 14%, respectively. Before targeted therapy, HER2+ tumours portended some of the worst prognoses, but the development of targeted therapies, such as trastuzumab, has resulted in a marked improvement in outcome. The triple-negative (TN) subtype (defined as ER−, PR− and HER2−) comprises 10–30% of all invasive breast cancers. However, this estimation varies dramatically depending on race/ethnicity (Carey *et al*, 2006; Stead *et al*, 2009). In general, across all ethnic groups, TN breast cancer is considered a subtype that can often confer a poor clinical outcome. However, no effective targeted therapies have been devised to date for this subtype.

Gene expression profiling has become an important research-screening tool in the identification and development of biomarkers that assess prognosis and prediction. Expression profiles have the potential to define cancer subtypes, prognosticate clinical outcome (i.e., recurrence of disease), predict response to specific therapies and identify critical oncogenic pathways (Huang *et al*, 2003). Investigations of signalling pathways and interactions indicated by gene signatures that are truly predictive of the clinical end points are necessary to understand the biology underlying this predictive value. When these gene signatures are combined with clinical and demographic factors, multiple forms of molecular (gene-based and protein) data can provide information that identifies unique tumour characteristics leading to individualised treatment strategies (van de Vijver *et al*, 2002; Nevins *et al*, 2003).

Although most array-based platforms utilise high-quality RNA prepared from frozen specimens, the newer DASL (cDNA-mediated Annealing, Selection, extension and Ligation) assay (Illumina Inc., San Diego, CA, USA) was specifically designed to profile small fragmented transcripts typically extracted from formalin-fixed, paraffin-embedded (FFPE) tissues because of the formalin-fixation process (Bibikova *et al*, 2004; Fan *et al*, 2004; Li *et al*, 2006; Abramovitz *et al*, 2008). The DASL platform utilised in this study is based upon multiplexed RT–PCR applied in a bead array-based format that enables mRNA transcript quantification from up to 512 genes using three independent probe sets per gene and can be used to expression profile up to 96 samples in a high-throughput manner (Bibikova *et al*, 2004; Fan *et al*, 2004).

In an initial DASL assay study, a more limited 231-gene cancer panel was used in order to profile both breast and colon cancer FFPE tumour samples. Cluster analysis was able to separate breast from colon tissue types and subsequently divide each tissue sample set into cancer *v*s normal (Bibikova *et al*, 2004). In a subsequent study, Bibikova *et al* (2004) demonstrated the utility of Illumina's commercially available 502-gene human cancer panel to profile prostate, colon, breast and lung, and were able to identify differentially regulated genes between cancerous and healthy FFPE tissues. More recently, Ravo *et al* (2008) have shown, on a limited set of 13 breast carcinomas, that the DASL assay used in conjunction with the HCP is reliable and sensitive and compared favourably with results obtained by microarray analysis of RNA extracted from the same frozen tumour samples. The DASL assay has also been used, in conjunction with a panel of 512 prostate-related genes, to identify RNA signatures in prostate cancer, including a 16-gene set that correlates with prostate cancer relapse (Bibikova *et al*, 2007).

In an effort to expand the utility of the DASL platform for breast cancer, we have designed a 512-gene custom human breast cancer panel (BCP) and used it to expression profile FFPE breast cancer tissue specimens currently not amenable for analysis on standard microarray platforms. Here we describe the validation of our 512-gene BCP and characterisation of breast cancer subtypes on the DASL bead array-based platform. We have identified a 30-gene set, which can be used to differentiate pathology-defined subtypes of breast cancer. Furthermore, several of these genes are prognostic of OS and RFS in publically available microarray data sets, indicating that they are valid biomarkers associated with the different outcomes related to the different breast cancer subtypes.

## Materials and methods

### Tumour tissue samples

Tumour samples from women with confirmed invasive carcinomas of the breast were obtained in the form of FFPE blocks. All archived FFPE tumour specimens were obtained from St Mary's Hospital (Montreal, Quebec, Canada) according to institutional guidelines. In total, we obtained 87 FFPE breast carcinomas that had previously been scored for the breast cancer markers, ER, PR and HER2 by immunohistochemistry (IHC) according to guidelines based on the ASCO/CAP recommendations for ER, PR and HER2 testing (ER/PR testing (http://www.cap.org/apps/docs/l aboratory_accreditation/summary_of_recommendations.pdf); HER2 testing (http://www.cap.org/apps/docs/c ommittees/immunohistochemistry/summary_of_recommendations.pdf)). In some IHC-equivocal cases of HER2 staining (IHC 2+), fluorescent *in situ* hybridisation (FISH) was used to confirm genomic amplification. The majority of the breast cancer tumour specimens used in this study were invasive ductal carcinomas (IDCs), including 2 tubular carcinomas and 1 invasive cribriform carcinoma; 2 of them had a sarcomatous component and 9 were mixed with invasive lobular carcinomas (ILCs). There were also 9 ILCs, the majority of which fell in the HR+ subtype. The FFPE blocks were archived 2 to 3 years before analysis.

### RNA extraction, purification and quality assessment

Three 5 *μ*m sections per FFPE block were used for RNA isolation. RNA deparaffinisation, extraction and purification were performed according to the High Pure FFPE RNA Micro Kit (Roche, Mannheim, Germany) protocol. RNA concentration and Å260/Å280 ratio were determined using the NanoDrop ND-1000 spectrophotometer (NanoDrop Technologies Inc., Wilmington, DE, USA). In addition, TaqMan (Applied Biosystems, Foster City, CA, USA) assays were performed on the ribosomal protein RPL13a gene in triplicate using 200 ng of RNA converted to cDNA to quantify usable copies of RNA molecules per sample. RNA quality was assessed by quantitative RT–PCR analysis of the housekeeping gene *RPL13A* (forward primer, 5′-GTACGCTGTGAAGGCATCAA-3′, and reverse primer, 5′-GTTGGTGTTCATCCGCTTG-3′) and the reactions were run on a HT7900 real-time PCR instrument (Applied Biosystems).

### Custom breast cancer DASL assay pool (DAP)

The custom breast cancer panel list of 512 candidate genes was submitted to Illumina for synthesis. The optimal oligonucleotide sequence for each of the 1536 gene probes was determined using an oligonucleotide-scoring algorithm. Illumina synthesised the oligonucleotide pool or DAP for the BCP for use with their 96-well Universal Array Matrix (UAM).

### DASL assay

In the procedure, biotinylated random nonamers (biotin-d(N)9) and oligo d(T)18 were used for cDNA synthesis and probes were designed such that they targeted unique regions of the gene without limiting the selection of the optimal probe to the 3′ ends of transcripts. Sequence-specific query oligonucleotides encompassing primer extension, ligation and universal PCR in highly multiplexed reactions (1536-plex), two-colour labelling and redundant (∼30-fold redundancy of each bead type) feature representation were used to probe up to three different exonic sites per gene. This protocol has been shown to increase assay sensitivity and reproducibility for quantitative detection of differential expression using RNA from FFPE tissues (Bibikova *et al*, 2004; Fan *et al*, 2004).

The DASL assay was performed on our 512-gene custom designed human BCP using 200 ng of input RNA at the Emory Biomarker Service Centre (Emory University, Atlanta, GA, USA). The manufacturer's instructions were followed without modification. Samples, including technical replicates (singleton to quadruplicate), were run in the DASL assay on two UAMs. The hybridised UAMs were scanned using the BeadStation 500 Instrument (Illumina Inc.).

### Data and statistical analysis

DASL transcript intensities were interpreted in GenomeStudio. Samples with insufficient signal-to-noise ratios (<3) were removed from subsequent analysis and the remaining samples were quartile normalised with plate scaling. Technical replicates within samples were average combined to create one signature per tumour.

**Figure 1:**
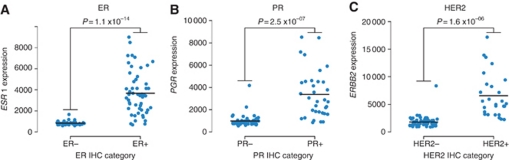
Expression of *ESR1*, *PGR* and *ERBB2* correspond to their respective pathology IHC staining status for ER, PR and HER2. Stripcharts showing the expression of (**A**) *ESR1*, (**B**) *PGR* and (**C**) *ERBB2* segregated by their respective negative and positive IHC categories. Gene-level data are displayed with the mean for each category denoted by the horizontal black bar and significance is assessed by a two-sided Welch's *t*-test.

The 30-gene set used to differentiate subtypes was determined using prediction analysis of microarrays (PAM) (Tibshirani *et al*, 2002), which was optimised by minimising the cross-validation training error (see Supplementary Table 2). Hierarchical clustering was conducted in R using the heatmap.2 package for each probe/gene-level data (Free Software Foundation, Boston, MA, USA), which was *Z*-score normalised with a dissimilarity metric based on Euclidean distance and an average algorithm for clustering. Significance for *ESR1*, *PGR* and *ERBB2* being differentially expressed between their respective IHC-positive and IHC-negative categories was assessed by Welch's *t*-test (Figure 1).

### Analysis of UNCCH-177 and NKI-295 cohorts

Data for the 295 patients from the Netherlands Cancer Institute (NKI-295) (van de Vijver *et al*, 2002) study were downloaded from the NKI website (http://bioinformatics.nki.nl/data.php), and data for the 177 patients from the University of North Carolina Chapel Hill (UNCCH-177) (Parker *et al*, 2009) were downloaded from Gene Expression Omnibus (GSE10886). Outcome data for the two studies were obtained for the associated Supplementary Information for the two publications. When clustering the data using the 30-gene set identified here, all probes for the given gene were used to cluster the data (Figure 3). Likewise, for the survival analysis of the 30 genes, all probes on the platforms for the NKI-295 and UNCCH-177 studies were analysed. One gene (*MLPH*) was not found on the NKI-295 platform. The 30 genes identified by PAM (Supplementary Table 2) were analysed for association with RFS and overall survival OS in the UNCCH-177 and NKI-295 cohorts using the Cox proportional hazards method implemented in R by the function ‘coxph’ of the ‘survival’ package (Supplementary Tables 3 and 4). All results of the survival analyses used in this manuscript have been included in Supplementary Tables 3 and 4. The raw PAM analysis spreadsheet identifying the 30 subtype differentiating genes in our cohort has also been provided as Supplementary Table 5.

All files used in the data analysis of this manuscript have been included in the Supplementary materials.

## Results

### Design of the human custom BCP for use in the DASL assay

In order to extend the utility of the DASL assay to the study of breast cancer utilising FFPE tumour specimens, we designed our own 512-gene BCP such that it incorporates previously identified signature genes from various breast cancer expression profiling studies that have been used in the intrinsic subclassification of breast tumours (Sørlie *et al*, 2001; Sotiriou *et al*, 2003), in prognosis (MammaPrint) (van’t Veer *et al*, 2002) and as predictors of outcome to treatment (Ayers *et al*, 2004; Jansen *et al*, 2005; Pawitan *et al*, 2005), including OncotypeDX (Paik *et al*, 2004). We also selected genes taken from published data on breast cancer (McLean *et al*, 2005) as well as additional genes that have been implicated in a number of cancer-related processes including proliferation, angiogenesis (Zhong *et al*, 2000; Heuze-Vourc’h *et al*, 2005), metastasis (Kang *et al*, 2003; Minn *et al*, 2005), DNA repair, apoptosis (Miller *et al*, 2005) and thrombosis (Kwaan *et al*, 2003). Additional cancer-related genes included in the panel are oncogenes, tumour-suppressor genes, cell cycle genes, telomerase-related genes, amplified genes, breast cancer stem cell genes and senescence-related genes (Brabletz *et al*, 2005; Collado *et al*, 2005; Dikmen *et al*, 2005; see Supplementary Table 1 for the list of genes that make up the BCP).

### Comparison to Illumina's human cancer panel

In order to compare data generated with the BCP to data generated with the HCP, we evaluated 174 RNA samples that were composed of 6 singletons, 82 duplicates, 4 triplicates and 1 quadruplicate, making for 98 technical replicate correlations across 87 tumour specimens. Technical replicates run on the BCP and HCP had average correlations of 0.9612 and 0.9613, respectively (Pearson's *r*^2^ correlation, see Supplementary Figures 1A and B). To further validate the BCP, we compared data generated with the BCP against data previously generated with the HCP using a set of 152 genes present in both panels (see Supplementary Figure 1C). We compared 81 FFPE tumour samples analysed on both panels and observed an average *r*^2^ correlation of 0.88 (see Supplementary Figure 1D). The lower correlation of technical replicates between panels, as compared with within panels, was most likely because of the intrinsic nature of competitive multiplexed PCR reactions using common primers with different sets of amplicons.

### Comparison of IHC data with DASL data for ER, PR and HER2

A total of 87 FFPE tumour specimens came from three major IHC subclasses and were composed of 24 ER−/PR−/HER2− (designated TN); 8 ER−/PR−/HER2+ (designated HER2+); 8 ER+/PR-/HER2+ 11 ER+/PR+/HER2+ 13 ER+/PR−/HER2− and 23 ER+/PR+/HER2− (designated HR+).

To determine whether the DASL assay yields comparable data to IHC data, the DASL assay gene intensity (expression) data were compared with the available IHC protein expression data for ER, PR and HER2 on the set of 87 tumour samples. For purposes of comparison of IHC data with DASL data, the IHC data provided for ER and PR were scored as either negative for staining (IHC staining <1% no expression detected) or as positive for staining (IHC staining ⩾1% which included weak, moderate or strong expression). For HER2, a score of 3+ was indicative of gene amplification (equivocal samples with a score of 2+ were tested in the FISH assay in order to rule out gene amplification). The tumours positive and negative for ER, PR and HER2 showed a significantly different level of expression for their respective genes *ESR1*, *PGR* and *ERBB2* (*P*<0.01, Welch's *t*-test; Figure 1). These data show that the concordance of DASL data with IHC data for all three receptors is very high, which is consistent with previous studies relating mRNA and IHC protein levels (Cronin *et al*, 2004; Gong *et al*, 2007).

Samples that stained positive for ER, PR and HER2 resulted in DASL mRNA average transcript fold changes in ESR1, PGR and ERBB2 (95% CI) of 4.46 (2.01–6.90), 3.41 (1.24–5.58) and 3.59 (1.40–5.77) greater than their respective IHC-negative tumours. Taken together, these data indicate concordance of DASL assay intensity with IHC-determined protein expression. Of interest, among HER2+ tumour samples, expression levels were found to be highest in the HR− group compared with the HR+ groups. This is consistent with previous work in which activated ER has been shown to downregulate expression of HER2 in human breast cancer cell lines (Russell and Hung, 1992).

### Analysis of the data by IHC subtype

**Figure 2:**
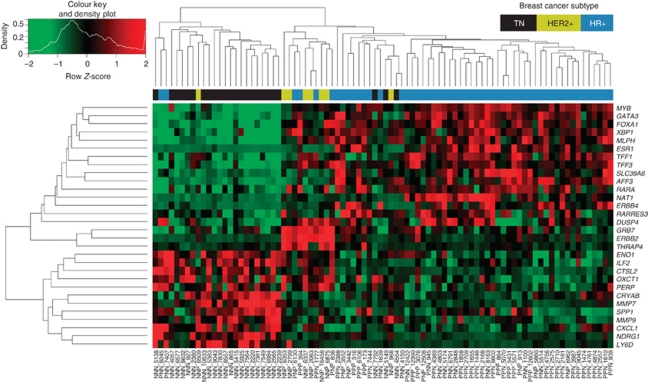
Montreal cohort of 87 patients hierarchically clustered across 30 genes predicative of immunohistochemical (IHC) breast cancer subtype. Prediction analysis of microarrays (PAM)-determined expression from 30 genes were indicative of IHC breast cancer subtype. Hierarchical clustering of patients (columns) and genes (rows) tends to segregate triple-negative (TN; indicated in black), HER2+ (indicated in yellow) and HR+ (indicated in blue) tumours. Red indicates upregulation and green downregulation of transcripts for genes labelled on the right. Gene transcript expression levels are *Z*-score normalised with a colour key indicated in the top left corner.

Unsupervised clustering of all samples was initially performed and clustering of the major subtypes, TN, HER2+ and HR+, is shown in Supplementary Figure 2. In order to define a set of genes that could be used to classify tumour samples, PAM (18) was used to identify 30 genes (Supplementary Table 2) that were indicative of the IHC breast cancer subtype in the Montreal cohort of 87 patients. Of these 30 genes, only 10 overlapped with Illumina's HCP. Additionally, 7 of the 30 genes overlapped with the PAM50 (*FOXA1*, *MLPH*, *ESR1*, *SLC39A6*, *NAT1*, *GRB7 and ERBB2*) (Parker *et al*, 2009) and 4 genes overlapped with OncotypeDX (*ESR1*, *ERBB2*, *CTSL2* and *GRB7*), 3 of which (underlined) were common to all three gene sets (see Supplementary Figure 3). Unsupervised hierarchical clustering across the 30 genes, as shown in Figure 2, tended to segregate TN, HER2+ and HR+ tumours.

**Figure 3:**
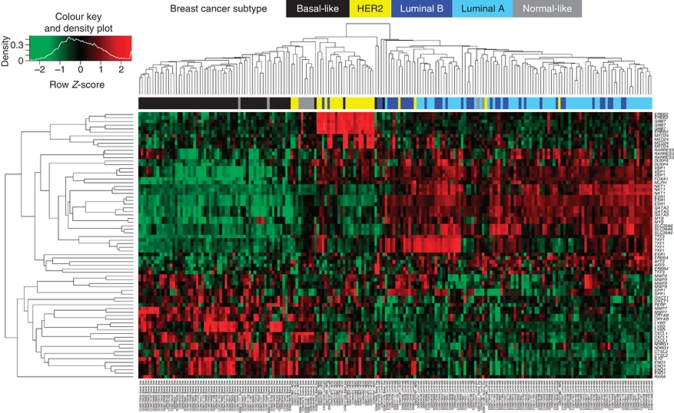
Hierarchical clustering of expression data from UNCCC cohort using probes from the 30 genes indicative of immunohistochemical (IHC) subtype in the Montreal cohort. Probes (rows) mapping to the 30 genes indicative of IHC subtype were used to hierarchically cluster patients (columns) in the Parker *et al* (2009) published microarray data (GEO data set GSE10886). These 30 genes segregate patients by ‘intrinsic subtype’. Red indicates upregulation and green downregulation of transcripts for probes labelled on the right. Probe transcript expression levels are *Z*-score normalised with a colour key indicated in the top left corner. Hierarchical clustering was conducted in R using the heatmap.2 package, with a dissimilarity metric based on Euclidean distance and an average algorithm for clustering.

This 30-gene set was then applied to the University of North Carolina Chapel Hill Lineberger Comprehensive Cancer Centre (UNCCH) published microarray data set originally described by Parker *et al* (2009) in order to determine if it would reproduce the delineation of intrinsic subtypes defined independently. Using the UNCCH cohort data set (*n*=177), unsupervised hierarchical clustering of the expression data using each probe for the 30 genes was performed and the heatmap is shown in Figure 3. The TN (basal-like), HER2+ and Normal-like clustered together whereas luminal B clusters were interspersed throughout the luminal A samples. Conversely, we also applied 33 genes of the PAM50 gene set, derived from fresh/frozen tissues and represented on the BCP, to our Montreal cohort with similar results (see Supplementary Figure 4). Thus, the 30-gene set applied to the UNCCH microarray data set was able to reproduce the delineation of intrinsic subtypes defined independently.

We then conducted an analysis of these 30 genes using the UNCCH data set as well as the NKI (*n*=295) published breast cancer microarray data set (van de Vijver *et al*, 2002), with respect to OS and RFS (Supplementary Figure 5 and Supplementary Tables 3 and 4). Several of the genes were significant by univariate Cox regression analysis in both data sets. Although this analysis does not identify or recommend a prognostic based on these genes, it does suggest that the genes that differentiate subtype found on our custom breast DASL panel are of prognostic importance in other cohorts, further inferring our ability to identify relevant expression patterns from FFPE material.

## Discussion

In order to extend the utility of the DASL assay with respect to microarray analysis of FFPE breast cancer tumour specimens, which represent a vast archive of well-characterised clinically annotated samples (Lewis *et al*, 2001), we have designed a 512-gene custom BCP for use in the DASL assay to expression profile mRNA transcripts using RNA extracted from FFPE tissues. The RNA isolated from FFPE tissue sources is highly degraded (average size ∼175 nt) and chemically modified (Masuda *et al*, 1999), and hence not amenable for conventional microarray analysis. In the DASL assay, because of the small size of the targeted gene sequence (∼50 nucleotides), the use of random primers in the cDNA synthesis and three independent probe sets per gene are important factors that enable the expression profiling of degraded RNAs on this bead array-based platform using a minimal amount of total RNA (200 ng per assay).

Although FFPE samples used in this study were 2 to 3 years old, others have shown that older samples, despite increasing RNA degradation with age (Cronin *et al*, 2004), can also be used successfully in the DASL assay (Bibikova *et al*, 2004). It has also been recently shown that the DASL assay can generate comparable expression profiling data when directly comparing FFPE and fresh/frozen tumour tissue pairs (Mittempergher *et al*, 2011). Taken together, there are many advantages to using the highly multiplexed DASL assay specifically for FFPE tumour samples that otherwise would not be easily amenable to other standard techniques such as RT–PCR, microarrays or RNA deep sequencing.

Given that we were limited to expression profiling of 512 genes on the DASL platform, we designed our own BCP such that 70% (360 out of 512) of the genes differed from Illumina's commercial HCP resulting in a panel composed of genes specifically related to breast cancer pathology including those from the OncotypeDX (Paik *et al*, 2004) and MammaPrint (van de Vijver *et al*, 2002) as well as angiogenic, metastatic and other breast cancer prognostic and predictive markers. Our BCP also includes 33 of the 50 PAM50 genes from a recently published breast cancer biomarker study (Parker *et al*, 2009), indicative of selection of highly relevant genes.

We first validated our BCP for use in the DASL assay through concordance of DASL data with ER, PR and HER2 IHC data as well as by comparison of 152 genes in common with the HCP. The data clearly demonstrate that degraded RNA isolated from FFPE breast tumour specimens can be expression profiled using our BCP in a highly reproducible, accurate and high-throughput manner on the bead array-based DASL platform. Results from such analysis are consistent with clinical pathology IHC markers for ER, PR and HER2 and comparable to results from fresh/frozen specimens on traditional microarray platforms as demonstrated on independent published microarray-generated data sets (see below).

We used PAM analysis and identified 30 genes that were indicative of IHC breast cancer subtype that segregate the pathology-defined subtypes in an unsupervised hierarchical analysis. The majority of these genes (19 out of 30) have previously been associated with particular breast cancer subtypes (Rouzier *et al*, 2005; Hu *et al*, 2006; Parker *et al*, 2009) except 11 genes, including *AFF3*, *DUSP4*, *ENO1*, *ERBB4*, *MMP7*, *MYB*, *PERP*, *RARA*, *SPP1*, *TFF1 and TFF3*. This is not surprising as even biomarker signatures developed for women with similar breast tumour characteristics on similar microarray platforms and using only fresh/frozen extracted mRNA may not share many genes in common. For example, the Rotterdam 76-gene signature (Wang *et al*, 2005) and the 70-gene MammaPrint signature (van de Vijver *et al*, 2002) in fact share not a single gene in common.

There were two genes, in addition to HER2, clearly overexpressed in HER2+ tumours, *GRB7* and *MED24*, which are both upstream of *ERBB2* on chromosome 17 and previously associated with the HER2+ subtype (Sørlie, 2004). Both *GRB7* and *MED24* have been found to be part of the smallest region of amplification (SRA), which extends from 34.73 to 35.48 Mb in HER2-amplified tumours but does not include TOP2A (Arriola *et al*, 2008). Another gene further upstream on chromosome 17, the *retinoic acid receptor* (*RARA*), was only found to be overexpressed in only some of the HER2+ tumour samples. The authors also found that in *HER2/TOP2A* co-amplified samples, the SRA that extended from 34.73 to 365.54 Mb also encompassed four additional genes including *RARA* (Arriola *et al*, 2008). These genes that are co-amplified along with HER2 may also play a role in cancer progression. For example, evidence in breast cancer cell lines suggests that *GRB7* may be involved in proliferation and that inhibiting both *HER2* and *GRB7* may enhance the inhibitory effect on cell growth (Pero *et al*, 2007).

When we applied our 30-gene set to published microarray data sets, we could reproduce the delineation of intrinsic subtypes defined independently. As it is well known that breast cancer subtypes have differing prognoses, we used these 30 genes to reanalyse two published breast cancer microarray studies, Parker *et al* (2009) and Van de Vijver *et al* (2002), with respect to OS and RFS. Seven genes were found prognostic of OS in both data sets and five of these seven were also prognostic of RFS. Several of these genes have previously been associated with the various subtypes. For example, *ESR1*, *NAT1*, *SLC39A6* and *XBP1* have been associated with the luminal A subtype (Sørlie, 2004), and *CSTL2* and *LYD6* are associated with the basal-like or TN subtype (Sørlie, 2004; Sørlie *et al*, 2006). In addition, we found that expression of *NDRG1*, which was prognostic of RFS but not OS, was mainly increased in TN tumours. This gene has been shown to be upregulated by hypoxic conditions and induced by the hypoxia-inducible factor HIF1A, the key transcription factor involved in the response of tumours to hypoxic conditions. Genes involved in the cellular response to hypoxia, including *NDRG1*, are associated with a significantly poorer prognosis in breast and ovarian cancers (Chi *et al*, 2006).

In this study we have shown that it is feasible to use degraded RNA prepared from FFPE breast tumour specimens, which represent the largest collection of well-annotated, clinical tumour samples that are readily available for conducting large retrospective studies, for expression profiling on the DASL platform. We have demonstrated the utility of conducting expression profiling of breast tumour samples using a custom selected set of genes to investigate pathological processes related to subtype. To exemplify this, we have herein identified markers that can be used to classify breast cancer into subtypes consistent with independent means of classification and shown these markers to be prognostic of OS and RFS. Finally, this study is further evidence that expression profiling of FFPE tumour banks can be productive, something that has now been demonstrated many times, for example, in a series of published studies of the Oncotype DX tests (Paik *et al*, 2004; Tang *et al*, 2011) and that analysis of FFPE samples on the DASL assay could lead to the discovery of biomarker sets that will have relevance in the clinical setting.

## Data Deposition

Gene Expression data are deposited in Gene Expression Omnibus (GEO) (http://www.ncbi.nlm.nih.gov/geo/) under series GSE17650.
