# Supplementary material for: Molecular characterisation of formalin-fixed paraffin-embedded (FFPE) breast tumour specimens using a custom 512-gene breast cancer bead array-based platform
Source: Br J Cancer. 2011 Nov 8;105(10):1574–81. doi: 10.1038/bjc.2011.355 (PMC3242517; doi:10.1038/bjc.2011.355)
Supplement: Supplementary Information [file bjc2011355x1.doc]

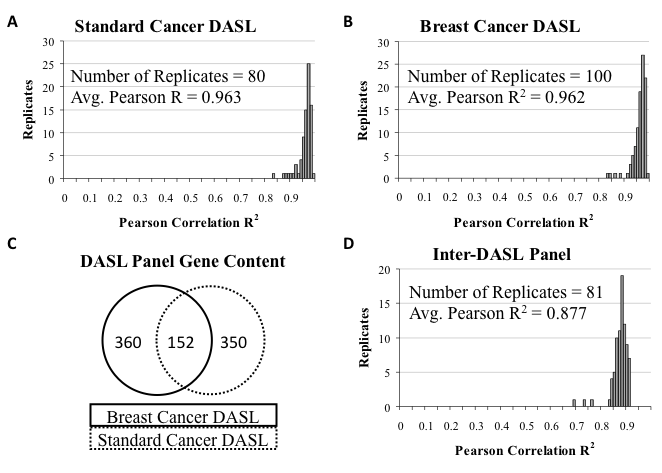


**Supplementary Figure 1. Histograms of DASL assay replicate reproducibility. A)** Human Cancer DASL Panel (HCP) reproducibility for 80 comparisons of RNA replicates across 502 genes covered by 1,488 probes yielded an average [95% Confidence Interval (CI)] Pearson r^2^ = 0.963 [0.882, 0.989]. **B)** Breast Cancer DASL Panel (BCP) reproducibility for a 100 comparisons of RNA replicates across 512 genes covered by 1,536 probes yielded an average [95% CI] Pearson r^2^ = 0.962 [0.872, 0.989]. **C)** Overlap of gene coverage between the HCP (dashed line) and the BCP (solid line) includes 152 genes. **D)** Correlation between 81 samples run on both the HCP and BCP yielded an average [95% CI] Pearson r^2^ = 0.877 [0.764, 0.914].


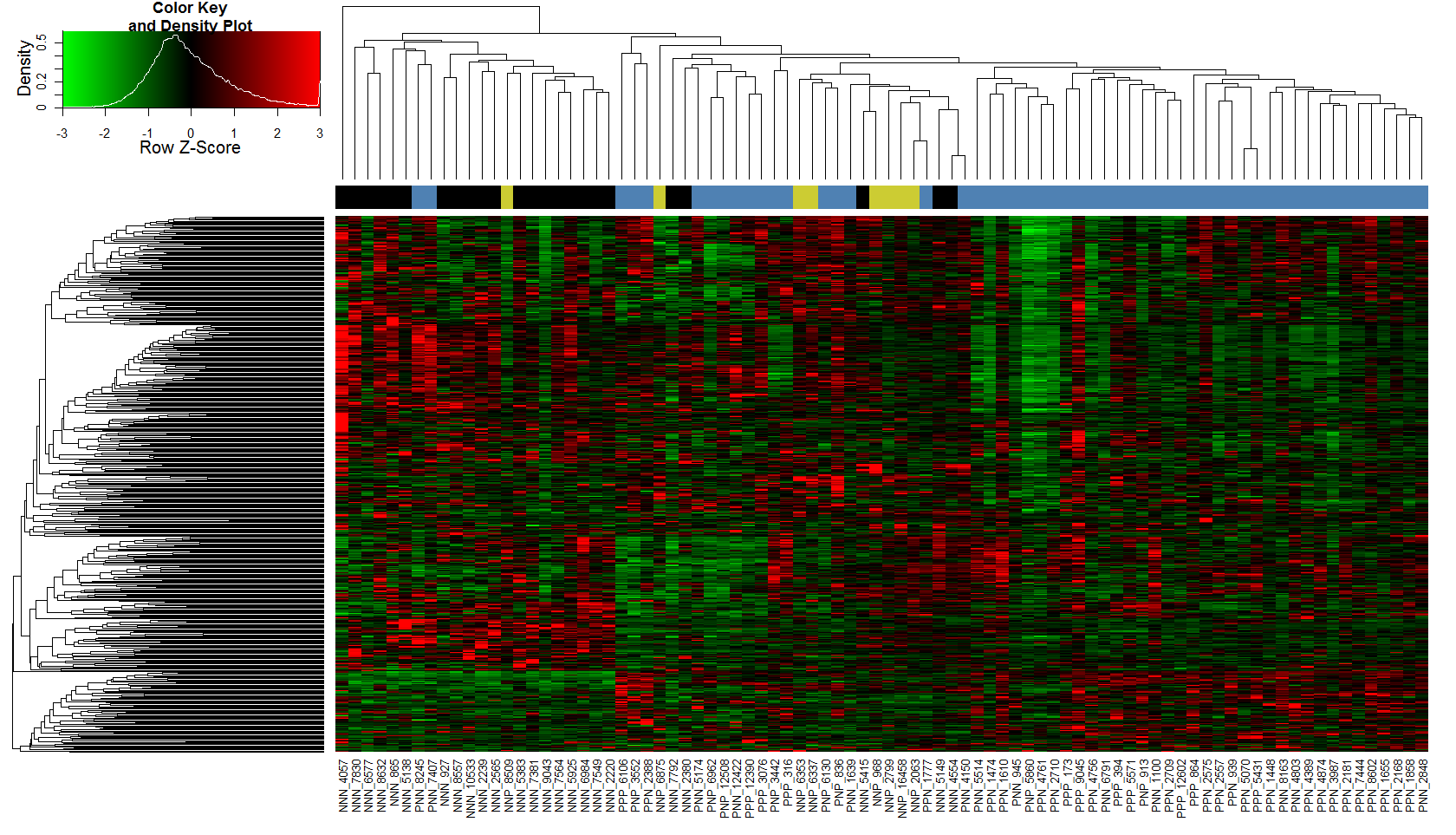


**Supplementary Figure 2. Unsupervised hierarchical clustering of 87 breast tumour samples from the Montreal cohort of patients.** Hierarchical clustering of patients (columns) and genes (rows) tends to segregate triple negative (TN; indicated in black), HER2+ (indicated in dark gray), and HR+ (indicated in light gray) tumours. Red indicates upregulation and green downregulation of transcripts for genes labelled on the right. Gene transcript expression levels are Z-score normalized with a colour key indicated in the top left corner. Hierarchical clustering was conducted in R using the heatmap.2 package, with a dissimilarity metric based on Euclidean distance and an average algorithm for clustering.


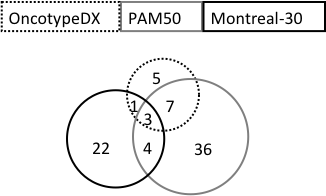


**Supplementary Figure 3.Venn Diagram ofGene overlap of the Montreal PAM30 genes identified as indicative of immunohistochemical (IHC) subtype with the PAM50 (Parker *et al*, 2009) and OncotypeDX® (Paik *et al,* 2004).**

**Supplementary Figure 4. Hierarchical clustering of Montreal cohort using 33/50 PAM50 genes on the custom breast-DASL panel.** Hierarchical clustering of patients (columns) and genes (rows) tends to segregate triple negative (TN; indicated in black), HER2+ (indicated in yellow), and HR+ (indicated in blue) tumours. Red indicates upregulation and green downregulation of transcripts for genes labelled on the right. Gene transcript expression levels are Z-score normalized with a colour key indicated in the top left corner. Hierarchical clustering was conducted in R using the heatmap.2 package, with a dissimilarity metric based on Euclidean distance and an average algorithm for clustering.


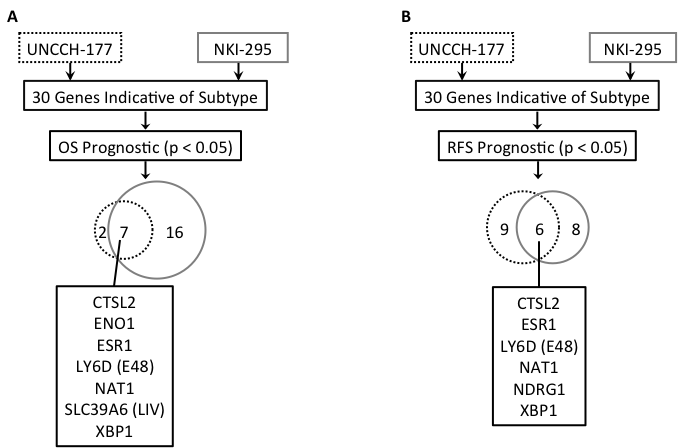


**Supplementary Figure 5.Genes prognostic of overall survival (OS) and recurrence free survival (RFS). A)** Cox proportional hazards analysis of the 30 genes predictive of IHC subtype in our data for association with OS and RFS in the UNCCH-177 (Parker *et al,* 2009;data set GSE10886) and NKI-295 (van de Vijver *et al,* 2002) data sets.Genes determined to be significant in prognosis of **A)** OS or **B)** RFS (p-value < 0.05) are shown in the Venn diagrams and listed in Supplementary Tables 3-4.

**Supplementary Table 1.** List of genes in the human custom breast cancer panel (BCP).

|  | **Gene identifier** | **Official Gene Symbol** | **Gene Name** | **Chromosomal Location** |
| --- | --- | --- | --- | --- |
| 1 | 5243 | ABCB1 | ATP-binding cassette, sub-family B (MDR/TAP), member 1 | 7q21.1 |
| 2 | 4363 | ABCC1 | ATP-binding cassette, sub-family C (CFTR/MRP), member 1 | 16p13.1 |
| 3 | 1244 | ABCC2 | ATP-binding cassette, sub-family C (CFTR/MRP), member 2 | 10q24 |
| 4 | 9429 | ABCG2 | ATP-binding cassette, sub-family G (WHITE), member 2 | 4q22 |
| 5 | 25 | ABL1 | v-abl Abelson murine leukemia viral oncogene homolog 1 | 9q34.1 |
| 6 | 35 | ACADS | acyl-Coenzyme A dehydrogenase, C-2 to C-3 short chain | 12q22-qter |
| 7 | 1636 | ACE | angiotensin I converting enzyme (peptidyl-dipeptidase A) 1 | 17q23 |
| 8 | 26027 | ACOT11 | thioesterase, adipose associated | 1p32.3 |
| 9 | 54 | ACP5 | acid phosphatase 5, tartrate resistant | 19p13.3-p13.2 |
| 10 | 60 | ACTB | actin, beta | 7p15-p12 |
| 11 | 133 | ADM | Adrenomedullin | 11p15.4 |
| 12 | 3899 | AFF3 | lymphoid nuclear protein related to AF4 | 2q11.2-q12 |
| 13 | 183 | AGT | angiotensinogen (serine (or cysteine) proteinase inhibitor, clade A (alpha-1 antiproteinase, antitrypsin), member 8) | 1q42-q43 |
| 14 | 205 | AK3 | adenylate kinase 3 | 1p31.3 |
| 15 | 207 | AKT1 | v-akt murine thymoma viral oncogene homolog 1 | 14q32.32 |
| 16 | 208 | AKT2 | v-akt murine thymoma viral oncogene homolog 2 | 19q13.1-q13.2 |
| 17 | 10000 | AKT3 | v-akt murine thymoma viral oncogene homolog 3 (protein kinase B, gamma) | 1q43-q44 |
| 18 | 214 | ALCAM | activated leukocyte cell adhesion molecule | 3q13.1 |
| 19 | 4329 | ALDH6A1 | aldehyde dehydrogenase 6 family, member A1 | 14q24.3 |
| 20 | 283 | ANG | angiogenin, ribonuclease, RNase A family, 5 | 14q11.1-q11.2 |
| 21 | 285 | ANGPT2 | angiopoietin 2 | 8p23.1 |
| 22 | 288 | ANK3 | ankyrin 3, node of Ranvier (ankyrin G) | 10q21 |
| 23 | 348 | APOE | apolipoprotein E | 19q13.2 |
| 24 | 374 | AREG | amphiregulin (schwannoma-derived growth factor) | 4q13-q21 |
| 25 | 397 | ARHGDIB | Rho GDP dissociation inhibitor (GDI) beta | 12p12.3 |
| 26 | 440 | ASNS | asparagine synthetase | 7q21.3 |
| 27 | 29028 | ATAD2 | PRO2000 protein | 8q24.13 |
| 28 | 22809 | ATF5 | activating transcription factor 5 | 19q13.3 |
| 29 | 506 | ATP5B | ATP synthase, H+ transporting, mitochondrial F1 complex, beta polypeptide | 12p13-qter |
| 30 | 5205 | ATP8B1 | ATPase, Class I, type 8B, member 1 | 18q21 |
| 31 | 545 | ATR | ataxia telangiectasia and Rad3 related | 3q22-q24 |
| 32 | 6790 | AURKA | serine/threonine kinase 6 | 20q13.2-q13.3 |
| 33 | 9212 | AURKB | aurora kinase B | 17p13.1 |
| 34 | 572 | BAD | BCL2-antagonist of cell death | 11q13.1 |
| 35 | 573 | BAG1 | BCL2-associated athanogene | 9p12 |
| 36 | 578 | BAK1 | BCL2-antagonist/killer 1 | 6p21.3 |
| 37 | 27113 | BBC3 | BCL2 binding component 3 | 19q13.3-q13.4 |
| 38 | 10286 | BCAS2 | breast carcinoma amplified sequence 2 | 1p21-p13.3 |
| 39 | 596 | BCL2 | B-cell CLL/lymphoma 2 | 18q21.3 |
| 40 | 598 | BCL2L1 | BCL2-like 1 | 20q11.21 |
| 41 | 79370 | BCL2L14 | apoptosis regulator BCL-G | 12p13-p12 |
| 42 | 8553 | BHLHB2 | basic helix-loop-helix domain containing, class B, 2 | 3p26 |
| 43 | 637 | BID | BH3 interacting domain death agonist | 22q11.1 |
| 44 | 638 | BIK | BCL2-interacting killer (apoptosis-inducing) | 22q13.31 |
| 45 | 274 | BIN1 | bridging integrator 1 | 2q14 |
| 46 | 332 | BIRC5 | baculoviral IAP repeat-containing 5 (survivin) | 17q25 |
| 47 | 641 | BLM | Bloom syndrome | 15q26.1 |
| 48 | 90427 | BMF | Bcl-2 modifying factor | 15q14 |
| 49 | 664 | BNIP3 | BCL2/adenovirus E1B 19kDa interacting protein 3 | 10q26.3 |
| 50 | 673 | BRAF | v-raf murine sarcoma viral oncogene homolog B1 | 7q34 |
| 51 | 672 | BRCA1 | breast cancer 1, early onset | 17q21 |
| 52 | 675 | BRCA2 | breast cancer 2, early onset | 13q12.3 |
| 53 | 7832 | BTG2 | BTG family, member 2 | 1q32 |
| 54 | 699 | BUB1 | BUB1 budding uninhibited by benzimidazoles 1 homolog (yeast) | 2q14 |
| 55 | 56672 | C11orf17 | chromosome 11 open reading frame 17 | 11p15.3 |
| 56 | 56946 | C11orf30 | chromosome 11 open reading frame 30 | 11q13.3 |
| 57 | 84075 | C14orf155 | hypothetical protein DKFZp434F1017 | 14q21.2 |
| 58 | 718 | C3 | complement component 3 | 19p13.3-p13.2 |
| 59 | 27092 | CACNG4 | calcium channel, voltage-dependent, gamma subunit 4 | 17q24 |
| 60 | 811 | CALR | calreticulin | 19p13.3-p13.2 |
| 61 | 835 | CASP2 | caspase 2, apoptosis-related cysteine protease (neural precursor cell expressed, developmentally down-regulated 2) | 7q34-q35 |
| 62 | 836 | CASP3 | caspase 3, apoptosis-related cysteine protease | 4q34 |
| 63 | 841 | CASP8 | caspase 8, apoptosis-related cysteine protease | 2q33-q34 |
| 64 | 891 | CCNB1 | cyclin B1 | 5q12 |
| 65 | 9133 | CCNB2 | cyclin B2 | 15q21.3 |
| 66 | 595 | CCND1 | cyclin D1 (PRAD1: parathyroid adenomatosis 1) | 11q13 |
| 67 | 894 | CCND2 | cyclin D2 | 12p13 |
| 68 | 898 | CCNE1 | cyclin E1 | 19q12 |
| 69 | 9134 | CCNE2 | cyclin E2 | 8q22.1 |
| 70 | 81669 | CCNL2 | cyclin L2 | 1p36.33 |
| 71 | 934 | CD24 | CD24 antigen (small cell lung carcinoma cluster 4 antigen) | 6q21 |
| 72 | 29126 | CD274 | programmed cell death 1 ligand 1 | 9p24 |
| 73 | 960 | CD44 | CD44 antigen (homing function and Indian blood group system) | 11p13 |
| 74 | 968 | CD68 | CD68 antigen | 17p13 |
| 75 | 983 | CDC2 | cell division cycle 2, G1 to S and G2 to M | 10q21.1 |
| 76 | 991 | CDC20 | CDC20 cell division cycle 20 homolog (S. cerevisiae) | 1p34.1 |
| 77 | 994 | CDC25B | cell division cycle 25B | 20p13 |
| 78 | 8476 | CDC42BPA | CDC42 binding protein kinase alpha (DMPK-like) | 1q42.11 |
| 79 | 23580 | CDC42EP4 | CDC42 effector protein (Rho GTPase binding) 4 | 17q24-q25 |
| 80 | 8318 | CDC45L | CDC45 cell division cycle 45-like (S. cerevisiae) | 22q11.21 |
| 81 | 990 | CDC6 | CDC6 cell division cycle 6 homolog (S. cerevisiae) | 17q21.3 |
| 82 | 999 | CDH1 | cadherin 1, type 1, E-cadherin (epithelial) | 16q22.1 |
| 83 | 1000 | CDH2 | cadherin 2, type 1, N-cadherin (neuronal) | 18q11.2 |
| 84 | 1001 | CDH3 | cadherin 3, type 1, P-cadherin (placental) | 16q22.1 |
| 85 | 1019 | CDK4 | cyclin-dependent kinase 4 | 12q14 |
| 86 | 1026 | CDKN1A | cyclin-dependent kinase inhibitor 1A (p21, Cip1) | 6p21.2 |
| 87 | 1027 | CDKN1B | cyclin-dependent kinase inhibitor 1B (p27, Kip1) | 12p13.1-p12 |
| 88 | 1028 | CDKN1C | cyclin-dependent kinase inhibitor 1C (p57, Kip2) | 11p15.5 |
| 89 | 1029 | CDKN2A | cyclin-dependent kinase inhibitor 2A (melanoma, p16, inhibits CDK4) | 9p21 |
| 90 | 1030 | CDKN2B | cyclin-dependent kinase inhibitor 2B (p15, inhibits CDK4) | 9p21 |
| 91 | 1058 | CENPA | centromere protein A, 17kDa | 2p24-p21 |
| 92 | 55839 | CENPN | uncharacterized bone marrow protein BM039 | 16q23.2 |
| 93 | 1066 | CES1 | carboxylesterase 1 (monocyte/macrophage serine esterase 1) | 16q13-q22.1 |
| 94 | 1081 | CGA | glycoprotein hormones, alpha polypeptide | 6q12-q21 |
| 95 | 1111 | CHEK1 | CHK1 checkpoint homolog (S. pombe) | 11q24-q24 |
| 96 | 11200 | CHEK2 | CHK2 checkpoint homolog (S. pombe) | 22q12.1 |
| 97 | 1117 | CHI3L2 | chitinase 3-like 2 | 1p13.3 |
| 98 | 1164 | CKS2 | CDC28 protein kinase regulatory subunit 2 | 9q22 |
| 99 | 1196 | CLK2 | CDC-like kinase 2 | 1q21 |
| 100 | 10256 | CNKSR1 | connector enhancer of KSR-like (Drosophila kinase suppressor of ras) | 1p35.3 |
| 101 | 80781 | COL18A1 | collagen, type XVIII, alpha 1 | 21q22.3 |
| 102 | 1284 | COL4A2 | collagen, type IV, alpha 2 | 13q34 |
| 103 | 1298 | COL9A2 | collagen, type IX, alpha 2 | 1p33-p32 |
| 104 | 1381 | CRABP1 | cellular retinoic acid binding protein 1 | 15q24 |
| 105 | 1410 | CRYAB | crystallin, alpha B | 11q22.3-q23.1 |
| 106 | 1438 | CSF2RA | colony stimulating factor 2 receptor, alpha, low-affinity (granulocyte-macrophage) | Xp22.32 and Yp11.3 |
| 107 | 1440 | CSF3 | colony stimulating factor 3 (granulocyte) | 17q11.2-q12 |
| 108 | 1460 | CSNK2B | casein kinase 2, beta polypeptide | 6p21.3 |
| 109 | 1469 | CST1 | cystatin SN | 20p11.21 |
| 110 | 1490 | CTGF | connective tissue growth factor | 6q23.1 |
| 111 | 1495 | CTNNA1 | catenin (cadherin-associated protein), alpha 1, 102kDa | 5q31 |
| 112 | 1499 | CTNNB1 | catenin (cadherin-associated protein), beta 1, 88kDa | 3p21 |
| 113 | 1515 | CTSL2 | cathepsin L2 | 9q22.2 |
| 114 | 6376 | CX3CL1 | chemokine (C-X3-C motif) ligand 1 | 16q13 |
| 115 | 2919 | CXCL1 | chemokine (C-X-C motif) ligand 1 (melanoma growth stimulating activity, alpha) | 4q21 |
| 116 | 3627 | CXCL10 | chemokine (C-X-C motif) ligand 10 | 4q21 |
| 117 | 9547 | CXCL14 | chemokine (C-X-C motif) ligand 14 | 5q31 |
| 118 | 7852 | CXCR4 | chemokine (C-X-C motif) receptor 4 | 2q21 |
| 119 | 1576 | CYP3A4 | cytochrome P450, family 3, subfamily A, polypeptide 4 | 7q21.1 |
| 120 | 3491 | CYR61 | cysteine-rich, angiogenic inducer, 61 | 1p31-p22 |
| 121 | 1603 | DAD1 | defender against cell death 1 | 14q11-q12 |
| 122 | 131566 | DCBLD2 | endothelial and smooth muscle cell-derived neuropilin-like protein | 3q12.2 |
| 123 | 1633 | DCK | deoxycytidine kinase | 4q13.3-q21.1 |
| 124 | 1642 | DDB1 | damage-specific DNA binding protein 1, 127kDa | 11q12-q13 |
| 125 | 50807 | DDEF1 | development and differentiation enhancing factor 1 | 8q24.1-q24.2 |
| 126 | 8560 | DEGS1 | degenerative spermatocyte homolog, lipid desaturase (Drosophila) | 1q42.12 |
| 127 | 23405 | DICER1 | Dicer1, Dcr-1 homolog (Drosophila) | 14q32.2 |
| 128 | 9787 | DLG7 | discs, large homolog 7 (Drosophila) | 14q22.2 |
| 129 | 3301 | - DNAJA1 | DnaJ (Hsp40) homolog, subfamily A, member 1 | 9p13-p12 |
| 130 | 1846 | DUSP4 | dual specificity phosphatase 4 | 8p12-p11 |
| 131 | 1869 | E2F1 | E2F transcription factor 1 | 20q11.2 |
| 132 | 1870 | E2F2 | E2F transcription factor 2 | 1p36 |
| 133 | 1915 | EEF1A1 | eukaryotic translation elongation factor 1 alpha 1 | 6q14.1 |
| 134 | 1942 | EFNA1 | ephrin-A1 | 1q21-q22 |
| 135 | 1948 | EFNB2 | ephrin-B2 | 13q33 |
| 136 | 1950 | EGF | epidermal growth factor (beta-urogastrone) | 4q25 |
| 137 | 1956 | EGFR | epidermal growth factor receptor (erythroblastic leukemia viral (v-erb-b) oncogene homolog, avian) | 7p12 |
| 138 | 1958 | EGR1 | early growth response 1 | 5q31.1 |
| 139 | 1963 | EIF1 | eukaryotic translation initiation factor 1 |  |
| 140 | 5610 | EIF2AK2 | eukaryotic translation initiation factor 2-alpha kinase 2 | 2p22-p21 |
| 141 | 1977 | EIF4E | eukaryotic translation initiation factor 4E | 4q21-q25 |
| 142 | 9669 | EIF5B | eukaryotic translation initiation factor 5B | 2p11.1-q11.1 |
| 143 | 2012 | EMP1 | epithelial membrane protein 1 | 12p12.3 |
| 144 | 2022 | ENG | endoglin (Osler-Rendu-Weber syndrome 1) | 9q33-q34.1 |
| 145 | 2023 | ENO1 | enolase 1, (alpha) | 1p36.3-p36.2 |
| 146 | 2033 | EP300 | E1A binding protein p300 | 22q13.2 |
| 147 | 57634 | EP400 | E1A binding protein p400 | 12q24.33 |
| 148 | 2034 | EPAS1 | endothelial PAS domain protein 1 | 2p21-p16 |
| 149 | 2056 | EPO | erythropoietin | 7q22 |
| 150 | 2057 | EPOR | erythropoietin receptor | 19p13.3-p13.2 |
| 151 | 3266 | ERAS | ES cell expressed Ras | Xp11.23 |
| 152 | 2064 | ERBB2 | v-erb-b2 erythroblastic leukemia viral oncogene homolog 2, neuro/glioblastoma derived oncogene homolog (avian) | 17q21.1 |
| 153 | 2065 | ERBB3 | v-erb-b2 erythroblastic leukemia viral oncogene homolog 3 (avian) | 12q13 |
| 154 | 2066 | ERBB4 | v-erb-a erythroblastic leukemia viral oncogene homolog 4 (avian) | 2q33.3-q34 |
| 155 | 2078 | ERG | v-ets erythroblastosis virus E26 oncogene like (avian) | 21q22.3 |
| 156 | 51614 | ERGIC3 | serologically defined breast cancer antigen 84 | 20pter-q12 |
| 157 | 9700 | ESPL1 | extra spindle poles like 1 (S. cerevisiae) | 8 |
| 158 | 2099 | ESR1 | estrogen receptor 1 | 6q25.1 |
| 159 | 2113 | ETS1 | v-ets erythroblastosis virus E26 oncogene homolog 1 (avian) | 11q23.3 |
| 160 | 2115 | ETV1 | ets variant gene 1 | 7p22 |
| 161 | 2120 | ETV6 | ets variant gene 6 (TEL oncogene) | 12p13 |
| 162 | 2131 | EXT1 | exostoses (multiple) 1 | 8q24.11-q24.13 |
| 163 | 2146 | EZH2 | enhancer of zeste homolog 2 (Drosophila) | 7q35-q36 |
| 164 | 2147 | F2 | coagulation factor II (thrombin) | 11p11-q12 |
| 165 | 2149 | F2R | coagulation factor II (thrombin) receptor | 5q13 |
| 166 | 2152 | F3 | coagulation factor III (thromboplastin, tissue factor) | 1p22-p21 |
| 167 | 355 | FAS | tumor necrosis factor receptor superfamily, member 6 | 10q24.1 |
| 168 | 2237 | FEN1 | flap structure-specific endonuclease 1 | 11q12 |
| 169 | 2246 | FGF1 | fibroblast growth factor 1 (acidic) | 5q31 |
| 170 | 2247 | FGF2 | fibroblast growth factor 2 (basic) | 4q26-q27 |
| 171 | 2249 | FGF4 | fibroblast growth factor 4 (heparin secretory transforming protein 1, Kaposi sarcoma oncogene) | 11q13.3 |
| 172 | 2260 | FGFR1 | fibroblast growth factor receptor 1 (fms-related tyrosine kinase 2, Pfeiffer syndrome) | 8p11.2-p11.1 |
| 173 | 2263 | FGFR2 | fibroblast growth factor receptor 2 (bacteria-expressed kinase, keratinocyte growth factor receptor, craniofacial dysostosis 1, Crouzon syndrome, Pfeiffer syndrome, Jackson-Weiss syndrome) | 10q26 |
| 174 | 2264 | FGFR4 | fibroblast growth factor receptor 4 | 5q35.1-qter |
| 175 | 2272 | FHIT | fragile histidine triad gene | 3p14.2 |
| 176 | 9158 | FIBP | fibroblast growth factor (acidic) intracellular binding protein | 11q13.1 |
| 177 | 2280 | FKBP1A | FK506 binding protein 1A, 12kDa | 20p13 |
| 178 | 2286 | FKBP2 | FK506 binding protein 2, 13kDa | 11q13.1-q13.3 |
| 179 | 2288 | FKBP4 | FK506 binding protein 4, 59kDa | 12p13.33 |
| 180 | 2314 | FLII | flightless I homolog (Drosophila) | 17p11.2 |
| 181 | 2317 | FLNB | filamin B, beta (actin binding protein 278) | 3p14.3 |
| 182 | 2321 | FLT1 | fms-related tyrosine kinase 1 (vascular endothelial growth factor/vascular permeability factor receptor) | 13q12 |
| 183 | 2322 | FLT3 | fms-related tyrosine kinase 3 | 13q12 |
| 184 | 2324 | FLT4 | fms-related tyrosine kinase 4 | 5q34-q35 |
| 185 | 2335 | FN1 | fibronectin 1 | 2q34 |
| 186 | 3169 | FOXA1 | forkhead box A1 | 14q12-q13 |
| 187 | 2296 | FOXC1 | forkhead box C1 | 6p25 |
| 188 | 2305 | FOXM1 | forkhead box M1 | 12p13 |
| 189 | 27315 | FRAG1 | FGF receptor activating protein 1 | 11p15.5 |
| 190 | 2475 | FRAP1 | FK506 binding protein 12-rapamycin associated protein 1 | 1p36.2 |
| 191 | 2530 | FUT8 | fucosyltransferase 8 (alpha (1,6) fucosyltransferase) | 14q24.3 |
| 192 | 8324 | FZD7 | frizzled homolog 7 (Drosophila) | 2q33 |
| 193 | 9846 | GAB2 | GRB2-associated binding protein 2 | 11q13.4 |
| 194 | 1647 | GADD45A | growth arrest and DNA-damage-inducible, alpha | 1p31.2-p31.1 |
| 195 | 2597 | GAPDH | glyceraldehyde-3-phosphate dehydrogenase | 12p13 |
| 196 | 2620 | GAS2 | growth arrest-specific 2 | 11p14.3-p15.2 |
| 197 | 2625 | GATA3 | GATA binding protein 3 | 10p15 |
| 198 | 2632 | GBE1 | glucan (1,4-alpha-), branching enzyme 1 (glycogen branching enzyme, Andersen disease, glycogen storage disease type IV) | 3p12.3 |
| 199 | 2637 | GBX2 | gastrulation brain homeo box 2 | 2q37 |
| 200 | 8836 | GGH | gamma-glutamyl hydrolase (conjugase, folylpolygammaglutamyl hydrolase) | 8q12.2 |
| 201 | 51659 | GINS2 | DNA replication complex GINS protein PSF2 | 16q24.1 |
| 202 | 8833 | GMPS | guanine monphosphate synthetase | 3q24 |
| 203 | 2781 | GNAZ | guanine nucleotide binding protein (G protein), alpha z polypeptide | 22q11.22 |
| 204 | 51280 | GOLPH2 | golgi phosphoprotein 2 | 9q21.33 |
| 205 | 9289 | GPR56 | G protein-coupled receptor 56 | 16q13 |
| 206 | 2879 | GPX4 | glutathione peroxidase 4 (phospholipid hydroperoxidase) | 19p13.3 |
| 207 | 2886 | GRB7 | growth factor receptor-bound protein 7 | 17q21.2 |
| 208 | 2934 | GSN | gelsolin (amyloidosis, Finnish type) | 9q33 |
| 209 | 2935 | GSPT1 | G1 to S phase transition 1 | 16p13.1 |
| 210 | 2936 | GSR | glutathione reductase | 8p21.1 |
| 211 | 2947 | GSTM3 | glutathione S-transferase M3 (brain) | 1p13.3 |
| 212 | 2950 | GSTP1 | glutathione S-transferase pi | 11q13 |
| 213 | 2967 | GTF2H3 | general transcription factor IIH, polypeptide 3, 34kDa | 12q24.31 |
| 214 | 51512 | GTSE1 | G-2 and S-phase expressed 1 | 22q13.2-q13.3 |
| 215 | 2990 | GUSB | glucuronidase, beta | 7q21.11 |
| 216 | 283120 | H19 | H19, imprinted maternally expressed untranslated mRNA | 11p15.5 |
| 217 | 3015 | H2AFZ | H2A histone family, member Z | 4q24 |
| 218 | 3054 | HCFC1 | host cell factor C1 (VP16-accessory protein) | Xq28 |
| 219 | 3065 | HDAC1 | histone deacetylase 1 | 1p34 |
| 220 | 3066 | HDAC2 | histone deacetylase 2 | 6q21 |
| 221 | 50810 | HDGFRP3 | hepatoma-derived growth factor, related protein 3 | 15q11.2 |
| 222 | 3091 | HIF1A | hypoxia-inducible factor 1, alpha subunit (basic helix-loop-helix transcription factor) | 14q21-q24 |
| 223 | 29923 | HIG2 | hypoxia-inducible protein 2 | 7q32.2 |
| 224 | 3159 | HMGA1 | high mobility group AT-hook 1 | 6p21 |
| 225 | 3202 | HOXA5 | homeo box A5 | 7p15-p14 |
| 226 | 10855 | HPSE | heparanase | 4q21.3 |
| 227 | 3265 | HRAS | v-Ha-ras Harvey rat sarcoma viral oncogene homolog | 11p15.5 |
| 228 | 57110 | HRASLS | HRAS-like suppressor | 3q29 |
| 229 | 3295 | HSD17B4 | hydroxysteroid (17-beta) dehydrogenase 4 | 5q21 |
| 230 | 3309 | HSPA5 | heat shock 70kDa protein 5 (glucose-regulated protein, 78kDa) | 9q33-q34.1 |
| 231 | 3397 | ID1 | inhibitor of DNA binding 1, dominant negative helix-loop-helix protein | 20q11 |
| 232 | 2537 | IFI6 | interferon, alpha-inducible protein (clone IFI-6-16) | 1p35 |
| 233 | 3458 | IFNG | interferon, gamma | 12q14 |
| 234 | 3479 | IGF1 | insulin-like growth factor 1 (somatomedin C) | 12q22-q23 |
| 235 | 3480 | IGF1R | insulin-like growth factor 1 receptor | 15q25-q26 |
| 236 | 3485 | IGFBP2 | insulin-like growth factor binding protein 2, 36kDa | 2q33-q34 |
| 237 | 3486 | IGFBP3 | insulin-like growth factor binding protein 3 | 7p13-p12 |
| 238 | 3487 | IGFBP4 | insulin-like growth factor binding protein 4 | 17q12-q21.1 |
| 239 | 3488 | IGFBP5 | insulin-like growth factor binding protein 5 | 2q33-q36 |
| 240 | 3490 | IGFBP7 | insulin-like growth factor binding protein 7 | 4q12 |
| 241 | 3586 | IL10 | interleukin 10 | 1q31-q32 |
| 242 | 3589 | IL11 | interleukin 11 | 19q13.3-q13.4 |
| 243 | 3592 | IL12A | interleukin 12A (natural killer cell stimulatory factor 1, cytotoxic lymphocyte maturation factor 1, p35) | 3p12-q13.2 |
| 244 | 3606 | IL18 | interleukin 18 (interferon-gamma-inducing factor) | 11q22.2-q22.3 |
| 245 | 3552 | IL1A | interleukin 1, alpha | 2q14 |
| 246 | 3553 | IL1B | interleukin 1, beta | 2q14 |
| 247 | 50604 | IL20 | interleukin 20 | 1q32 |
| 248 | 3569 | IL6 | interleukin 6 (interferon, beta 2) | 7p21 |
| 249 | 3576 | IL8 | interleukin 8 | 4q13-q21 |
| 250 | 3608 | ILF2 | interleukin enhancer binding factor 2, 45kDa | 1q22 |
| 251 | 3611 | ILK | integrin-linked kinase | 11p15.5-p15.4 |
| 252 | 3658 | IREB2 | iron-responsive element binding protein 2 | 15q24.1 |
| 253 | 3659 | IRF1 | interferon regulatory factor 1 | 5q31.1 |
| 254 | 3685 | ITGAV | integrin, alpha V (vitronectin receptor, alpha polypeptide, antigen CD51) | 2q31-q32 |
| 255 | 23421 | ITGB3BP | integrin beta 3 binding protein (beta3-endonexin) | 1p31.3 |
| 256 | 182 | JAG1 | jagged 1 (Alagille syndrome) | 20p12.1-p11.23 |
| 257 | 3791 | KDR | kinase insert domain receptor (a type III receptor tyrosine kinase) | 4q11-q12 |
| 258 | 10112 | KIF20A | kinesin family member 20A | 5q31 |
| 259 | 9493 | KIF23 | kinesin family member 23 | 15q22.31 |
| 260 | 11004 | KIF2C | kinesin family member 2C | 1p34.1 |
| 261 | 3815 | KIT | v-kit Hardy-Zuckerman 4 feline sarcoma viral oncogene homolog | 4q11-q12 |
| 262 | 1316 | KLF6 | core promoter element binding protein | 10p15 |
| 263 | 10403 | KNTC2 | kinetochore associated 2 | 18p11.31 |
| 264 | 3838 | KPNA2 | karyopherin alpha 2 (RAG cohort 1, importin alpha 1) | 17q23.1-q23.3 |
| 265 | 3845 | KRAS | v-Ki-ras2 Kirsten rat sarcoma 2 viral oncogene homolog | 12p12.1 |
| 266 | 3848 | KRT1 | keratin 1 (epidermolytic hyperkeratosis) | 12q12-q13 |
| 267 | 3860 | KRT13 | keratin 13 | 17q12-q21.2 |
| 268 | 3872 | KRT17 | keratin 17 | 17q12-q21 |
| 269 | 3880 | KRT19 | keratin 19 | 17q21.2 |
| 270 | 3852 | KRT5 | keratin 5 (epidermolysis bullosa simplex, Dowling-Meara/Kobner/Weber-Cockayne types) | 12q12-q13 |
| 271 | 3854 | KRT6B | keratin 6B | 12q12-q13 |
| 272 | 3897 | L1CAM | L1 cell adhesion molecule (hydrocephalus, stenosis of aqueduct of Sylvius 1, MASA (mental retardation, aphasia, shuffling gait and adducted thumbs) syndrome, spastic paraplegia 1) | Xq28 |
| 273 | 3918 | LAMC2 | laminin, gamma 2 | 1q25-q31 |
| 274 | 55353 | LAPTM4B | lysosomal associated protein transmembrane 4 beta | 8q22.1 |
| 275 | 3939 | LDHA | lactate dehydrogenase A | 11p15.4 |
| 276 | 4000 | LMNA | lamin A/C | 1q21.2-q21.3 |
| 277 | 4015 | LOX | lysyl oxidase | 5q23.2 |
| 278 | 4017 | LOXL2 | lysyl oxidase-like 2 | 8p21.3-p21.2 |
| 279 | 987 | LRBA | LPS-responsive vesicle trafficking, beach and anchor containing | 4q31.23 |
| 280 | 4036 | LRP2 | low density lipoprotein-related protein 2 | 2q24-q31 |
| 281 | 4049 | LTA | lymphotoxin alpha (TNF superfamily, member 1) | 6p21.3 |
| 282 | 4052 | LTBP1 | latent transforming growth factor beta binding protein 1 | 2p22-p21 |
| 283 | 8581 | LY6D | lymphocyte antigen 6 complex, locus D | 8q24-qter |
| 284 | 4085 | MAD2L1 | MAD2 mitotic arrest deficient-like 1 (yeast) | 4q27 |
| 285 | 114569 | MAL2 | mal, T-cell differentiation protein 2 |  |
| 286 | 6416 | MAP2K4 | mitogen-activated protein kinase kinase 4 | 17p11.2 |
| 287 | 5594 | MAPK1 | mitogen-activated protein kinase 1 | 22q11.21 |
| 288 | 1432 | MAPK14 | mitogen-activated protein kinase 14 | 6p21.3-p21.2 |
| 289 | 4137 | MAPT | microtubule-associated protein tau | 17q21.1 |
| 290 | 10150 | MBNL2 | muscleblind-like 2 (Drosophila) | 13q32.2 |
| 291 | 4170 | MCL1 | myeloid cell leukemia sequence 1 (BCL2-related) | 1q21 |
| 292 | 4171 | MCM2 | MCM2 minichromosome maintenance deficient 2, mitotin (S. cerevisiae) | 3q21 |
| 293 | 4175 | MCM6 | MCM6 minichromosome maintenance deficient 6 (MIS5 homolog, S. pombe) (S. cerevisiae) | 2q21 |
| 294 | 4193 | MDM2 | Mdm2, transformed 3T3 cell double minute 2, p53 binding protein (mouse) | 12q14.3-q15 |
| 295 | 9862 | MED24 | mediator complex subunit 24 | 17q21.2 |
| 296 | 9833 | MELK | maternal embryonic leucine zipper kinase | 9p13.1 |
| 297 | 4240 | MFGE8 | milk fat globule-EGF factor 8 protein | 15q25 |
| 298 | 4259 | MGST3 | microsomal glutathione S-transferase 3 | 1q23 |
| 299 | 4288 | MKI67 | antigen identified by monoclonal antibody Ki-67 | 10q25-qter |
| 300 | 79682 | MLF1IP | KSHV latent nuclear antigen interacting protein 1 | 4q35.1 |
| 301 | 4292 | MLH1 | mutL homolog 1, colon cancer, nonpolyposis type 2 (E. coli) | 3p21.3 |
| 302 | 8028 | MLLT10 | myeloid/lymphoid or mixed-lineage leukemia (trithorax homolog, Drosophila); translocated to, 10 | 10p12 |
| 303 | 79083 | MLPH | melanophilin | 2q37.3 |
| 304 | 4312 | MMP1 | matrix metalloproteinase 1 (interstitial collagenase) | 11q22.3 |
| 305 | 4320 | MMP11 | matrix metalloproteinase 11 (stromelysin 3) | 22q11.23 |
| 306 | 4313 | MMP2 | matrix metalloproteinase 2 (gelatinase A, 72kDa gelatinase, 72kDa type IV collagenase) | 16q13-q21 |
| 307 | 4314 | MMP3 | matrix metalloproteinase 3 (stromelysin 1, progelatinase) | 11q22.3 |
| 308 | 4316 | MMP7 | matrix metallopeptidase 7 (matrilysin, uterine) | 11q21-q22 |
| 309 | 4318 | MMP9 | matrix metalloproteinase 9 (gelatinase B, 92kDa gelatinase, 92kDa type IV collagenase) | 20q11.2-q13.1 |
| 310 | 6183 | MRPS12 | mitochondrial ribosomal protein S12 | 19q13.1-q13.2 |
| 311 | 4436 | MSH2 | mutS homolog 2, colon cancer, nonpolyposis type 1 (E. coli) | 2p22-p21 |
| 312 | 2956 | MSH6 | mutS homolog 6 (E. coli) | 2p16 |
| 313 | 4582 | MUC1 | mucin 1, transmembrane | 1q21 |
| 314 | 4602 | MYB | v-myb myeloblastosis viral oncogene homolog (avian) | 6q22-q23 |
| 315 | 4605 | MYBL2 | v-myb myeloblastosis viral oncogene homolog (avian)-like 2 | 20q13.1 |
| 316 | 4609 | MYC | v-myc myelocytomatosis viral oncogene homolog (avian) | 8q24.12-q24.13 |
| 317 | 11143 | MYST2 | MYST histone acetyltransferase 2 | 17q21.32 |
| 318 | 9 | NAT1 | N-acetyltransferase 1 (arylamine N-acetyltransferase) | 8p23.1-p21.3 |
| 319 | 8202 | NCOA3 | nuclear receptor coactivator 3 | 20q12 |
| 320 | 4693 | NDP | Norrie disease (pseudoglioma) | Xp11.4 |
| 321 | 10397 | NDRG1 | N-myc downstream regulated gene 1 | 8q24.3 |
| 322 | 10763 | NES | nestin | 1q23.1 |
| 323 | 10725 | NFAT5 | nuclear factor of activated T-cells 5, tonicity-responsive | 16q22.1 |
| 324 | 4800 | NFYA | nuclear transcription factor Y, alpha | 6p21.3 |
| 325 | 4804 | NGFR | nerve growth factor receptor (TNFR superfamily, member 16) | 17q21-q22 |
| 326 | 4830 | NME1 | non-metastatic cells 1, protein (NM23A) expressed in | 17q21.3 |
| 327 | 4853 | NOTCH2 | Notch homolog 2 (Drosophila) | 1p13-p11 |
| 328 | 4855 | NOTCH4 | Notch homolog 4 (Drosophila) | 6p21.3 |
| 329 | 27020 | NPTN | stromal cell derived factor receptor 1 | 15q22 |
| 330 | 4886 | NPY1R | neuropeptide Y receptor Y1 | 4q31.3-q32 |
| 331 | 4893 | NRAS | neuroblastoma RAS viral (v-ras) oncogene homolog | 1p13.2 |
| 332 | 8829 | NRP1 | neuropilin 1 | 10p12 |
| 333 | 4916 | NTRK3 | neurotrophic tyrosine kinase, receptor, type 3 | 15q25 |
| 334 | 51203 | NUSAP1 | nucleolar and spindle associated protein 1 | 15q14 |
| 335 | 23595 | ORC3L | origin recognition complex, subunit 3-like (yeast) | 6q14.3-q16.1 |
| 336 | 23594 | ORC6L | origin recognition complex, subunit 6 homolog-like (yeast) | 16q12 |
| 337 | 5019 | OXCT1 | 3-oxoacid CoA transferase | 5p13 |
| 338 | 5033 | P4HA1 | procollagen-proline, 2-oxoglutarate 4-dioxygenase (proline 4-hydroxylase), alpha polypeptide I | 10q21.3-q23.1 |
| 339 | 143 | PARP4 | ADP-ribosyltransferase (NAD+; poly (ADP-ribose) polymerase)-like 1 | 13q11 |
| 340 | 5037 | PBP | prostatic binding protein | 12q24.23 |
| 341 | 5111 | PCNA | proliferating cell nuclear antigen | 20pter-p12 |
| 342 | 5127 | PCTK1 | PCTAIRE protein kinase 1 | Xp11.3-p11.23 |
| 343 | 9141 | PDCD5 | programmed cell death 5 | 19q12-q13.1 |
| 344 | 5154 | PDGFA | platelet-derived growth factor alpha polypeptide | 7p22 |
| 345 | 5155 | PDGFB | platelet-derived growth factor beta polypeptide (simian sarcoma viral (v-sis) oncogene homolog) | 22q13.1 |
| 346 | 10611 | PDLIM5 | LIM protein (similar to rat protein kinase C-binding enigma) | 4q22 |
| 347 | 10630 | PDPN | lung type-I cell membrane-associated glycoprotein | 1p36 |
| 348 | 93210 | PERLD1 | per1-like domain containing 1 | 17q12 |
| 349 | 64065 | PERP | PERP, TP53 apoptosis effector | 6q24 |
| 350 | 5214 | PFKP | phosphofructokinase, platelet | 10p15.3-p15.2 |
| 351 | 5230 | PGK1 | phosphoglycerate kinase 1 | Xq13 |
| 352 | 5241 | PGR | progesterone receptor | 11q22-q23 |
| 353 | 5290 | PIK3CA | phosphoinositide-3-kinase, catalytic, alpha polypeptide | 3q26.3 |
| 354 | 5295 | PIK3R1 | phosphoinositide-3-kinase, regulatory subunit, polypeptide 1 (p85 alpha) | 5q13.1 |
| 355 | 9088 | PKMYT1 | membrane-associated tyrosine- and threonine-specific cdc2-inhibitory kinase | 16p13.3 |
| 356 | 22925 | PLA2R1 | phospholipase A2 receptor 1, 180kDa | 2q23-q24 |
| 357 | 5327 | PLAT | plasminogen activator, tissue | 8p12 |
| 358 | 5328 | PLAU | plasminogen activator, urokinase | 10q24 |
| 359 | 5329 | PLAUR | plasminogen activator, urokinase receptor | 19q13 |
| 360 | 5347 | PLK1 | polo-like kinase (Drosophila) | 16p12.3 |
| 361 | 5378 | PMS1 | PMS1 postmeiotic segregation increased 1 (S. cerevisiae) | 2q31.1 |
| 362 | 5395 | PMS2 | PMS2 postmeiotic segregation increased 2 (S. cerevisiae) | 7p22.2 |
| 363 | 10721 | POLQ | polymerase (DNA directed), theta | 3q13.33 |
| 364 | 84513 | PPAPDC1B | HTPAP protein | 8p11.23 |
| 365 | 5469 | PPARBP | PPAR binding protein | 17q12-q21.1 |
| 366 | 5501 | PPP1CC | protein phosphatase 1, catalytic subunit, gamma isoform | 12q24.1-q24.2 |
| 367 | 5518 | PPP2R1A | protein phosphatase 2 (formerly 2A), regulatory subunit A (PR 65), alpha isoform | 19q13.41 |
| 368 | 5531 | PPP4C | protein phosphatase 4 (formerly X), catalytic subunit | 16p12-16p11 |
| 369 | 23532 | PRAME | preferentially expressed antigen in melanoma | 22q11.22 |
| 370 | 9055 | PRC1 | protein regulator of cytokinesis 1 | 15q26.1 |
| 371 | 10549 | PRDX4 | peroxiredoxin 4 | Xp22.13 |
| 372 | 5591 | PRKDC | protein kinase, DNA-activated, catalytic polypeptide | 8q11 |
| 373 | 5635 | PRPSAP1 | phosphoribosyl pyrophosphate synthetase-associated protein 1 | 17q24-q25 |
| 374 | 5701 | PSMC2 | proteasome (prosome, macropain) 26S subunit, ATPase, 2 | 7q22.1-q22.3 |
| 375 | 5708 | PSMD2 | proteasome (prosome, macropain) 26S subunit, non-ATPase, 2 | 3q27.3 |
| 376 | 5713 | PSMD7 | proteasome (prosome, macropain) 26S subunit, non-ATPase, 7 (Mov34 homolog) | 16q23-q24 |
| 377 | 9791 | PTDSS1 | phosphatidylserine synthase 1 | 8q22 |
| 378 | 5728 | PTEN | phosphatase and tensin homolog (mutated in multiple advanced cancers 1) | 10q23.3 |
| 379 | 5743 | PTGS2 | prostaglandin-endoperoxide synthase 2 (prostaglandin G/H synthase and cyclooxygenase) | 1q25.2-q25.3 |
| 380 | 5764 | PTN | pleiotrophin (heparin binding growth factor 8, neurite growth-promoting factor 1) | 7q33-q34 |
| 381 | 8073 | PTP4A2 | protein tyrosine phosphatase type IVA, member 2 | 1p35 |
| 382 | 11031 | RAB31 | RAB31, member RAS oncogene family | 18p11.3 |
| 383 | 51560 | RAB6B | RAB6B, member RAS oncogene family | 3q22.1 |
| 384 | 7879 | RAB7A | RAB7A, member RAS oncogene family | 3q21.3 |
| 385 | 5879 | RAC1 | ras-related C3 botulinum toxin substrate 1 | 7p22 |
| 386 | 29127 | RACGAP1 | Rac GTPase activating protein 1 | 12q13.12 |
| 387 | 5885 | RAD21 | RAD21 homolog (S. pombe) | 8q24 |
| 388 | 5901 | RAN | RAN, member RAS oncogene family | 6p21 |
| 389 | 5910 | RAP1GDS1 | RAP1, GTP-GDP dissociation stimulator 1 | 4q23-q25 |
| 390 | 5914 | RARA | retinoic acid receptor, alpha | 17q21 |
| 391 | 5920 | RARRES3 | retinoic acid receptor responder (tazarotene induced) 3 | 11q23 |
| 392 | 5925 | RB1 | retinoblastoma 1 (including osteosarcoma) | 13q14.2 |
| 393 | 5949 | RBP3 | retinol binding protein 3, interstitial | 10q11.2 |
| 394 | 116362 | RBP7 | retinoid binding protein 7 | 1p36.22 |
| 395 | 85004 | RERG | RAS-like, estrogen-regulated, growth-inhibitor | 12p13.1 |
| 396 | 5984 | RFC4 | replication factor C (activator 1) 4, 37kDa | 3q27 |
| 397 | 6001 | RGS10 | regulator of G-protein signalling 10 | 10q25 |
| 398 | 6009 | RHEB | Ras homolog enriched in brain | 7q36 |
| 399 | 388 | RHOB | ras homolog gene family, member B | 2pter-p12 |
| 400 | 57381 | RHOJ | ras homolog gene family, member J | 14q23.2 |
| 401 | 25907 | RIS1 | Ras-induced senescence 1 | 3p21.3 |
| 402 | 26994 | RNF11 | ring finger protein 11 | 1pter-p22.1 |
| 403 | 6045 | RNF2 | ring finger protein 2 | 1q25.3 |
| 404 | 56475 | RPRM | candidate mediator of the p53-dependent G2 arrest | 2q24.1 |
| 405 | 862 | RUNX1T1 | core-binding factor, runt domain, alpha subunit 2; translocated to, 1; cyclin D-related | 8q22 |
| 406 | 864 | RUNX3 | runt-related transcription factor 3 | 1p36 |
| 407 | 6281 | S100A10 | S100 calcium binding protein A10 (annexin II ligand, calpactin I, light polypeptide (p11)) | 1q21 |
| 408 | 6275 | S100A4 | S100 calcium binding protein A4 (calcium protein, calvasculin, metastasin, murine placental homolog) | 1q21 |
| 409 | 92304 | SCGB3A1 | secretoglobin, family 3A, member 1 | 5q35-qter |
| 410 | 6340 | SCNN1G | sodium channel, nonvoltage-gated 1, gamma | 16p12 |
| 411 | 9997 | SCO2 | SCO cytochrome oxidase deficient homolog 2 (yeast) | 22q13.33 |
| 412 | 57758 | SCUBE2 | signal peptide, CUB domain, EGF-like 2 | 11p15.3 |
| 413 | 6385 | SDC4 | syndecan 4 (amphiglycan, ryudocan) | 20q12 |
| 414 | 6388 | SDF2 | stromal cell-derived factor 2 | 17q11.2 |
| 415 | 8991 | SELENBP1 | selenium binding protein 1 | 1q21-q22 |
| 416 | 6405 | SEMA3F | sema domain, immunoglobulin domain (Ig), short basic domain, secreted, (semaphorin) 3F | 3p21.3 |
| 417 | 12 | SERPINA3 | serine (or cysteine) proteinase inhibitor, clade A (alpha-1 antiproteinase, antitrypsin), member 3 | 14q32.1 |
| 418 | 1992 | SERPINB1 | serine (or cysteine) proteinase inhibitor, clade B (ovalbumin), member 1 | 6p25 |
| 419 | 5055 | SERPINB2 | serine (or cysteine) proteinase inhibitor, clade B (ovalbumin), member 2 | 18q21.3 |
| 420 | 5054 | SERPINE1 | serine (or cysteine) proteinase inhibitor, clade E (nexin, plasminogen activator inhibitor type 1), member 1 | 7q21.3-q22 |
| 421 | 5176 | SERPINF1 | serpin peptidase inhibitor, clade F | 17p13.1 |
| 422 | 6422 | SFRP1 | secreted frizzled-related protein 1 | 8p12-p11.1 |
| 423 | 8487 | SIP1 | survival of motor neuron protein interacting protein 1 | 14q13 |
| 424 | 6494 | SIPA1 | signal-induced proliferation-associated gene 1 | 11q13 |
| 425 | 6563 | SLC14A1 | solute carrier family 14 (urea transporter), member 1 (Kidd blood group) | 18q11-q12 |
| 426 | 6513 | SLC2A1 | solute carrier family 2 (facilitated glucose transporter), member 1 | 1p35-p31.3 |
| 427 | 6515 | SLC2A3 | solute carrier family 2 (facilitated glucose transporter), member 3 | 12p13.3 |
| 428 | 10559 | SLC35A1 | solute carrier family 35 (CMP-sialic acid transporter), member A1 | 6q15 |
| 429 | 25800 | SLC39A6 | solute carrier family 39 (zinc transporter), member 6 | 18q12.2 |
| 430 | 9353 | SLIT2 | slit homolog 2 (Drosophila) | 4p15.2 |
| 431 | 6597 | SMARCA4 | SWI/SNF related, matrix associated, actin dependent regulator of chromatin, subfamily a, member 4 | 19p13.2 |
| 432 | 6615 | SNAI1 | snail homolog 1 (Drosophila) | 20q13.1-q13.2 |
| 433 | 6591 | SNAI2 | snail homolog 2 (Drosophila) | 8q11 |
| 434 | 6623 | SNCG | synuclein, gamma (breast cancer-specific protein 1) | 10q23.2-q23.3 |
| 435 | 6696 | SPP1 | secreted phosphoprotein 1 (osteopontin, bone sialoprotein I, early T-lymphocyte activation 1) | 4q21-q25 |
| 436 | 6713 | SQLE | squalene epoxidase | 8q24.1 |
| 437 | 57522 | SRGAP1 | SLIT-ROBO Rho GTPase activating protein 1 | 12q14.1 |
| 438 | 6776 | STAT5A | signal transducer and activator of transcription 5A | 17q11.2 |
| 439 | 8614 | STC2 | stanniocalcin 2 | 5q35.2 |
| 440 | 6794 | STK11 | serine/threonine kinase 11 (Peutz-Jeghers syndrome) | 19p13.3 |
| 441 | 10494 | STK25 | serine/threonine kinase 25 (STE20 homolog, yeast) | 2q37.3 |
| 442 | 6788 | STK3 | serine/threonine kinase 3 (STE20 homolog, yeast) | 8q22.2 |
| 443 | 11198 | SUPT16H | suppressor of Ty 16 homolog (S. cerevisiae) | 14q11.2 |
| 444 | 6840 | SVIL | supervillin | 10p11.2 |
| 445 | 10579 | TACC2 | transforming, acidic coiled-coil containing protein 2 | 10q26 |
| 446 | 4072 | TACSTD1 | tumor-associated calcium signal transducer 1 | 2p21 |
| 447 | 4070 | TACSTD2 | tumor-associated calcium signal transducer 2 | 1p32-p31 |
| 448 | 6876 | TAGLN | transgelin | 11q23.2 |
| 449 | 9519 | TBPL1 | TBP-like 1 | 6q22.1-q22.3 |
| 450 | 6925 | TCF4 | transcription factor 4 | 18q21.1 |
| 451 | 6935 | TCF8 | transcription factor 8 (represses interleukin 2 expression) | 10p11.2 |
| 452 | 6997 | TDGF1 | teratocarcinoma-derived growth factor 1 | 3p21.31 |
| 453 | 7011 | TEP1 | telomerase-associated protein 1 | 14q11.2 |
| 454 | 7015 | TERT | telomerase reverse transcriptase | 5p15.33 |
| 455 | 7022 | TFAP2C | transcription factor AP-2 gamma (activating enhancer binding protein 2 gamma) | 20q13.2 |
| 456 | 7023 | TFAP4 | transcription factor AP-4 (activating enhancer binding protein 4) | 16p13 |
| 457 | 7031 | TFF1 | trefoil factor 1 (breast cancer, estrogen-inducible sequence expressed in) | 21q22.3 |
| 458 | 7033 | TFF3 | trefoil factor 3 (intestinal) | 21q22.3 |
| 459 | 7035 | TFPI | tissue factor pathway inhibitor (lipoprotein-associated coagulation inhibitor) | 2q31-q32.1 |
| 460 | 7037 | TFRC | transferrin receptor (p90, CD71) | 3q26.2-qter |
| 461 | 7039 | TGFA | transforming growth factor, alpha | 2p13 |
| 462 | 7040 | TGFB1 | transforming growth factor, beta 1 (Camurati-Engelmann disease) | 19q13.1 |
| 463 | 7043 | TGFB3 | transforming growth factor, beta 3 | 14q24 |
| 464 | 7057 | THBS1 | thrombospondin 1 | 15q15 |
| 465 | 7060 | THBS4 | thrombospondin 4 | 5q13 |
| 466 | 7069 | THRSP | thyroid hormone responsive (SPOT14 homolog, rat) | 11q13.5 |
| 467 | 7076 | TIMP1 | tissue inhibitor of metalloproteinase 1 (erythroid potentiating activity, collagenase inhibitor) | Xp11.3-p11.23 |
| 468 | 7077 | TIMP2 | tissue inhibitor of metalloproteinase 2 | 17q25 |
| 469 | 7078 | TIMP3 | tissue inhibitor of metalloproteinase 3 (Sorsby fundus dystrophy, pseudoinflammatory) | 22q12.3 |
| 470 | 7079 | TIMP4 | tissue inhibitor of metalloproteinase 4 | 3p25 |
| 471 | 7083 | TK1 | thymidine kinase 1, soluble | 17q23.2-q25.3 |
| 472 | 9874 | TLK1 | tousled-like kinase 1 | 2q31.1 |
| 473 | 58986 | TMEM8 | transmembrane protein 8 (five membrane-spanning domains) | 16p13.3 |
| 474 | 7113 | TMPRSS2 | transmembrane protease, serine 2 | 21q22.3 |
| 475 | 7124 | TNF | tumor necrosis factor (TNF superfamily, member 2) | 6p21.3 |
| 476 | 8795 | TNFRSF10B | tumor necrosis factor receptor superfamily, member 10b | 8p22-p21 |
| 477 | 8793 | TNFRSF10D | tumor necrosis factor receptor superfamily, member 10d, decoy with truncated death domain | 8p21 |
| 478 | 8743 | TNFSF10 | tumor necrosis factor (ligand) superfamily, member 10 | 3q26 |
| 479 | 8600 | TNFSF11 | tumor necrosis factor (ligand) superfamily, member 11 | 13q14 |
| 480 | 8741 | TNFSF13 | tumor necrosis factor (ligand) superfamily, member 13 | 17p13.1 |
| 481 | 9966 | TNFSF15 | tumor necrosis factor (ligand) superfamily, member 15 | 9q32 |
| 482 | 7150 | TOP1 | topoisomerase (DNA) I | 20q12-q13.1 |
| 483 | 7153 | TOP2A | topoisomerase (DNA) II alpha 170kDa | 17q21-q22 |
| 484 | 7157 | TP53 | tumor protein p53 (Li-Fraumeni syndrome) | 17p13.1 |
| 485 | 23650 | TRIM29 | tripartite motif-containing 29 | 11q22-q23 |
| 486 | 9319 | TRIP13 | thyroid hormone receptor interactor 13 | 5p15.33 |
| 487 | 7248 | TSC1 | tuberous sclerosis 1 | 9q34 |
| 488 | 8848 | TSC22D1 | transforming growth factor beta-stimulated protein TSC-22 | 13q14 |
| 489 | 10103 | TSPAN1 | tetraspan 1 | 1p34.1 |
| 490 | 27075 | TSPAN13 | transmembrane 4 superfamily member 13 | 7p21.2 |
| 491 | 10098 | TSPAN5 | transmembrane 4 superfamily member 9 | 4q23 |
| 492 | 7283 | TUBG1 | tubulin, gamma 1 | 17q21 |
| 493 | 7291 | TWIST1 | twist homolog 1 (acrocephalosyndactyly 3; Saethre-Chotzen syndrome) (Drosophila) | 7p21.2 |
| 494 | 29089 | UBE2T | HSPC150 protein similar to ubiquitin-conjugating enzyme | 1q32.1 |
| 495 | 54963 | UCKL1 | uridine kinase-like 1 | 20q13.33 |
| 496 | 29128 | UHRF1 | ubiquitin-like, containing PHD and RING finger domains, 1 | 19p13.3 |
| 497 | 23326 | USP22 | ubiquitin specific protease 22 | 17p11.2 |
| 498 | 7412 | VCAM1 | vascular cell adhesion molecule 1 | 1p32-p31 |
| 499 | 7422 | VEGF | vascular endothelial growth factor | 6p12 |
| 500 | 79679 | VTCN1 | immune costimulatory protein B7-H4 | 1p12 |
| 501 | 7450 | VWF | von Willebrand factor | 12p13.3 |
| 502 | 7471 | WNT1 | wingless-type MMTV integration site family, member 1 | 12q13 |
| 503 | 7486 | WRN | Werner syndrome | 8p12-p11.2 |
| 504 | 7494 | XBP1 | X-box binding protein 1 | 22pter-q13 |
| 505 | 7534 | YWHAZ | tyrosine 3-monooxygenase/tryptophan 5-monooxygenase activation protein, zeta polypeptide | 8q23.1 |
| 506 | 26137 | ZBTB20 | zinc finger protein 288 | 3q13.2 |
| 507 | 678 | ZFP36L2 | zinc finger protein 36, C3H type-like 2 | 2p22.3-p21 |
| 508 | 7764 | ZNF217 | zinc finger protein 217 | 20q13.2 |
| 509 | 27246 | ZNF364 | zinc finger protein 364 | 1q21.2 |
| 510 | 79759 | ZNF668 | hypothetical protein FLJ13479 | 16p11.2 |
| 511 | 10320 | ZNFN1A1 | zinc finger protein, subfamily 1A, 1 (Ikaros) | 7p13-p11.1 |
| 512 | 7589 | ZSCAN21 | zinc finger and SCAN domain containing 21 | 7q22.1 |

**Supplementary Table 2.** 30 genes indicative of immunohistochemical (IHC) breast cancer subtype in the Montreal cohort of 87 patients identified by Prediction Analysis of Microarrays (PAM).

| **Gene identifier** | **Official Gene Symbol** | **Gene Name** | **Chromosomal Location** |
| --- | --- | --- | --- |
| 3899 | AFF3 | lymphoid nuclear protein related to AF4 | 2q11.2-q12 |
| 1410 | CRYAB | crystallin, alpha B | 11q22.3-q23.1 |
| 1515 | CTSL2 | cathepsin L2 | 9q22.2 |
| 2919 | CXCL1 | chemokine (C-X-C motif) ligand 1 (melanoma growth stimulating activity, alpha) | 4q21 |
| 1846 | DUSP4 | dual specificity phosphatase 4 | 8p12-p11 |
| 2023 | ENO1 | enolase 1, (alpha) | 1p36.3-p36.2 |
| 2064 | ERBB2 | v-erb-b2 erythroblastic leukemia viral oncogene homolog 2, neuro/glioblastoma derived oncogene homolog (avian) | 17q21.1 |
| 2066 | ERBB4 | v-erb-a erythroblastic leukemia viral oncogene homolog 4 (avian) | 2q33.3-q34 |
| 2099 | ESR1 | estrogen receptor 1 | 6q25.1 |
| 3169 | FOXA1 | forkhead box A1 | 14q12-q13 |
| 2625 | GATA3 | GATA binding protein 3 | 10p15 |
| 2886 | GRB7 | growth factor receptor-bound protein 7 | 17q21.2 |
| 3608 | ILF2 | interleukin enhancer binding factor 2, 45kDa | 1q22 |
| 8581 | LY6D | lymphocyte antigen 6 complex, locus D | 8q24-qter |
| 9862 | MED24 | mediator complex subunit 24 | 17q21.2 |
| 79083 | MLPH | melanophilin | 2q37.3 |
| 4316 | MMP7 | matrix metallopeptidase 7 (matrilysin, uterine) | 11q21-q22 |
| 4318 | MMP9 | matrix metalloproteinase 9 (gelatinase B, 92kDa gelatinase, 92kDa type IV collagenase) | 20q11.2-q13.1 |
| 4602 | MYB | v-myb myeloblastosis viral oncogene homolog (avian) | 6q22-q23 |
| 9 | NAT1 | N-acetyltransferase 1 (arylamine N-acetyltransferase) | 8p23.1-p21.3 |
| 10397 | NDRG1 | N-myc downstream regulated gene 1 | 8q24.3 |
| 5019 | OXCT1 | 3-oxoacid CoA transferase | 5p13 |
| 64065 | PERP | TP53 apoptosis effector | 6q24 |
| 5914 | RARA | retinoic acid receptor, alpha | 17q21 |
| 5920 | RARRES3 | retinoic acid receptor responder (tazarotene induced) 3 | 11q23 |
| 25800 | SLC39A6 | solute carrier family 39 (zinc transporter), member 6 | 18q12.2 |
| 6696 | SPP1 | secreted phosphoprotein 1 (osteopontin, bone sialoprotein I, early T-lymphocyte activation 1) | 4q21-q25 |
| 7031 | TFF1 | trefoil factor 1 (breast cancer, estrogen-inducible sequence expressed in) | 21q22.3 |
| 7033 | TFF3 | trefoil factor 3 (intestinal) | 21q22.3 |
| 7494 | XBP1 | X-box binding protein 1 | 22pter-q13 |

**Supplementary Table 3.**Cox univariate regression analysis of our 30 gene set in the NKI-295 data set. The coefficient (coef), expected coefficient (exp coef), standard error of the coefficient (se coef), z-statistic, and *p*-value are shown for both overall survival (OS) and recurrence free survival (RFS).

|  | **OS analysis** | | | | | **RFS analysis** | | | | |
| --- | --- | --- | --- | --- | --- | --- | --- | --- | --- | --- |
| **Probe** | **coef** | **exp coef** | **se coef** | **z.stat** | **p.value** | **coef** | **exp coef** | **se coef** | **z.stat** | **p.value** |
| **CRYAB_NM_001885** | 1.77 | 5.89 | 0.51 | 3.47 | 0.000527421 | 0.43 | 1.54 | 0.46 | 0.95 | 0.341947506 |
| **CTSL2_NM_001333** | 3.49 | 32.63 | 0.55 | 6.31 | 2.72E-10 | 2.24 | 9.35 | 0.47 | 4.72 | 2.38E-06 |
| **DUSP4_NM_001394** | -1.62 | 0.20 | 0.40 | -4.09 | 4.35E-05 | -1.24 | 0.29 | 0.34 | -3.65 | 0.000259376 |
| **E48_X82693** | 1.41 | 4.09 | 0.33 | 4.32 | 1.55E-05 | 0.97 | 2.65 | 0.30 | 3.24 | 0.001187091 |
| **ENO1_NM_001428** | 1.87 | 6.50 | 0.80 | 2.33 | 0.019981622 | 0.97 | 2.64 | 0.60 | 1.63 | 0.102656865 |
| **ERBB2_NM_004448** | 0.92 | 2.50 | 0.44 | 2.08 | 0.037752711 | 0.64 | 1.90 | 0.36 | 1.76 | 0.077952498 |
| **ERBB4_NM_005235** | -1.85 | 0.16 | 0.45 | -4.15 | 3.38E-05 | -1.34 | 0.26 | 0.39 | -3.46 | 0.000542085 |
| **ESR1_NM_000125** | -1.59 | 0.20 | 0.31 | -5.07 | 4.02E-07 | -1.07 | 0.34 | 0.27 | -3.99 | 6.64E-05 |
| **GATA3_NM_002051** | -0.51 | 0.60 | 0.24 | -2.13 | 0.033309461 | -0.31 | 0.74 | 0.23 | -1.36 | 0.175030255 |
| **GRB7_NM_005310** | 1.39 | 4.00 | 0.40 | 3.43 | 0.000614487 | 0.94 | 2.55 | 0.34 | 2.77 | 0.005656238 |
| **GRO1_NM_001511** | -0.45 | 0.64 | 0.54 | -0.83 | 0.408396259 | -0.37 | 0.69 | 0.44 | -0.84 | 0.402844005 |
| **HNF3A_Contig48177_RC** | -1.06 | 0.35 | 0.80 | -1.33 | 0.184202837 | -1.02 | 0.36 | 0.65 | -1.56 | 0.118745881 |
| **HNF3A_NM_004496** | -0.87 | 0.42 | 0.29 | -2.99 | 0.002823773 | -0.44 | 0.64 | 0.27 | -1.63 | 0.102064052 |
| **ILF2_NM_004515** | 4.37 | 79.02 | 0.89 | 4.89 | 1.01E-06 | 2.31 | 10.12 | 0.72 | 3.22 | 0.001297113 |
| **LAF4_NM_002285** | -0.51 | 0.60 | 0.61 | -0.83 | 0.404674774 | -0.29 | 0.75 | 0.50 | -0.57 | 0.566201228 |
| **LIV_1_NM_012319** | -0.83 | 0.43 | 0.40 | -2.11 | 0.034919949 | -0.32 | 0.72 | 0.34 | -0.97 | 0.333662972 |
| **MMP7_Z11887** | 0.81 | 2.25 | 0.38 | 2.16 | 0.031133158 | 0.60 | 1.83 | 0.31 | 1.97 | 0.048392744 |
| **MMP9_NM_004994** | 1.19 | 3.29 | 0.43 | 2.75 | 0.005994316 | 1.16 | 3.20 | 0.36 | 3.22 | 0.001295463 |
| **MYB_NM_005375** | -2.43 | 0.09 | 0.47 | -5.16 | 2.49E-07 | -1.55 | 0.21 | 0.40 | -3.91 | 9.09E-05 |
| **NAT1_NM_000662** | -1.68 | 0.19 | 0.38 | -4.38 | 1.20E-05 | -1.07 | 0.34 | 0.31 | -3.41 | 0.000654098 |
| **NDRG1_NM_006096** | 3.14 | 23.03 | 0.54 | 5.85 | 5.03E-09 | 1.71 | 5.52 | 0.47 | 3.67 | 0.000245566 |
| **OXCT_NM_000436** | 2.35 | 10.52 | 0.96 | 2.46 | 0.014052873 | 1.06 | 2.89 | 0.75 | 1.42 | 0.156578596 |
| **RARA_NM_000964** | 0.23 | 1.26 | 0.70 | 0.33 | 0.742207216 | 0.82 | 2.28 | 0.57 | 1.44 | 0.151155032 |
| **RARRES3_NM_004585** | -0.32 | 0.73 | 0.53 | -0.60 | 0.550056095 | -0.28 | 0.76 | 0.44 | -0.62 | 0.532747707 |
| **SPP1_NM_000582** | 1.23 | 3.43 | 0.47 | 2.62 | 0.008815381 | 0.75 | 2.12 | 0.38 | 1.99 | 0.046173911 |
| **TFF1_NM_003225** | -1.24 | 0.29 | 0.35 | -3.51 | 0.000450345 | -0.45 | 0.64 | 0.28 | -1.59 | 0.112814672 |
| **TFF3_NM_003226** | -1.14 | 0.32 | 0.35 | -3.28 | 0.001046619 | -0.21 | 0.81 | 0.30 | -0.70 | 0.482893597 |
| **XBP1_NM_005080** | -0.96 | 0.38 | 0.36 | -2.65 | 0.00805284 | -0.79 | 0.45 | 0.34 | -2.32 | 0.020247383 |

**Supplementary Table 4.**Cox univariate regression analysis of our 30 gene set in the UNCCH-177 data set. The coefficient (coef), expected coefficient (exp coef), standard error of the coefficient (se coef), z-statistic, and *p*-value are shown for both overall survival (OS) and recurrence free survival (RFS).

|  | **OS analysis** | | | | | **RFS analysis** | | | | |
| --- | --- | --- | --- | --- | --- | --- | --- | --- | --- | --- |
| **Probe** | **coef** | **exp coef** | **se coef** | **z.stat** | **p.value** | **coef** | **exp coef** | **se coef** | **z.stat** | **p.value** |
| **AFF3_12436** | -0.63 | 0.53 | 0.23 | -2.81 | 5.02E-03 | -0.69 | 0.50 | 0.21 | -3.34 | 8.45E-04 |
| **AFF3_13133** | -0.27 | 0.77 | 0.21 | -1.28 | 2.01E-01 | -0.28 | 0.76 | 0.19 | -1.46 | 1.45E-01 |
| **CRYAB_1345** | 0.11 | 1.11 | 0.11 | 0.98 | 3.29E-01 | 0.15 | 1.16 | 0.10 | 1.47 | 1.42E-01 |
| **CRYAB_15231** | 0.12 | 1.13 | 0.12 | 1.02 | 3.09E-01 | 0.19 | 1.20 | 0.10 | 1.79 | 7.41E-02 |
| **CTSL2_15611** | 0.23 | 1.26 | 0.12 | 1.98 | 4.77E-02 | 0.30 | 1.35 | 0.11 | 2.85 | 4.32E-03 |
| **CTSL2_18996** | 0.26 | 1.29 | 0.12 | 2.08 | 3.71E-02 | 0.32 | 1.38 | 0.11 | 2.90 | 3.72E-03 |
| **CXCL1_8105** | 0.16 | 1.17 | 0.14 | 1.15 | 2.49E-01 | 0.28 | 1.32 | 0.12 | 2.30 | 2.16E-02 |
| **CXCL1_9935** | 0.12 | 1.13 | 0.10 | 1.28 | 2.01E-01 | 0.21 | 1.23 | 0.09 | 2.46 | 1.37E-02 |
| **CXCL1_15797** | 0.12 | 1.13 | 0.09 | 1.40 | 1.62E-01 | 0.18 | 1.19 | 0.08 | 2.32 | 2.01E-02 |
| **DUSP4_6156** | -0.04 | 0.96 | 0.11 | -0.36 | 7.20E-01 | -0.13 | 0.88 | 0.10 | -1.36 | 1.73E-01 |
| **DUSP4_9421** | -0.02 | 0.98 | 0.20 | -0.13 | 8.98E-01 | -0.20 | 0.82 | 0.19 | -1.06 | 2.91E-01 |
| **ENO1_1005** | 0.27 | 1.31 | 0.19 | 1.44 | 1.49E-01 | 0.37 | 1.45 | 0.17 | 2.20 | 2.78E-02 |
| **ENO1_12004** | 0.36 | 1.43 | 0.18 | 1.98 | 4.79E-02 | 0.46 | 1.58 | 0.16 | 2.87 | 4.11E-03 |
| **ENO1_13921** | 0.35 | 1.42 | 0.20 | 1.80 | 7.18E-02 | 0.49 | 1.63 | 0.17 | 2.82 | 4.87E-03 |
| **ENO1_20094** | 0.29 | 1.33 | 0.19 | 1.49 | 1.36E-01 | 0.41 | 1.51 | 0.17 | 2.40 | 1.64E-02 |
| **ERBB2_784** | -0.13 | 0.88 | 0.10 | -1.30 | 1.95E-01 | -0.17 | 0.84 | 0.10 | -1.77 | 7.67E-02 |
| **ERBB2_5366** | 0.05 | 1.05 | 0.14 | 0.37 | 7.15E-01 | -0.02 | 0.98 | 0.14 | -0.15 | 8.81E-01 |
| **ERBB2_14017** | -0.03 | 0.97 | 0.12 | -0.25 | 8.05E-01 | -0.10 | 0.90 | 0.12 | -0.83 | 4.07E-01 |
| **ERBB4_1767** | -0.22 | 0.80 | 0.28 | -0.81 | 4.16E-01 | -0.49 | 0.61 | 0.27 | -1.82 | 6.85E-02 |
| **ERBB4_10121** | -0.21 | 0.81 | 0.14 | -1.47 | 1.41E-01 | -0.22 | 0.80 | 0.13 | -1.70 | 9.00E-02 |
| **ESR1_4558** | -0.10 | 0.90 | 0.05 | -2.09 | 3.67E-02 | -0.16 | 0.85 | 0.05 | -3.28 | 1.03E-03 |
| **ESR1_7486** | -0.12 | 0.89 | 0.05 | -2.41 | 1.59E-02 | -0.17 | 0.85 | 0.05 | -3.44 | 5.71E-04 |
| **ESR1_15713** | -0.11 | 0.90 | 0.05 | -2.15 | 3.16E-02 | -0.16 | 0.85 | 0.05 | -3.33 | 8.55E-04 |
| **ESR1_20432** | -0.71 | 0.49 | 0.33 | -2.12 | 3.40E-02 | -0.95 | 0.39 | 0.28 | -3.38 | 7.23E-04 |
| **FOXA1_308** | -0.07 | 0.94 | 0.06 | -1.17 | 2.43E-01 | -0.13 | 0.88 | 0.05 | -2.58 | 9.79E-03 |
| **GATA3_9467** | -0.11 | 0.89 | 0.08 | -1.45 | 1.47E-01 | -0.16 | 0.85 | 0.07 | -2.23 | 2.56E-02 |
| **GATA3_14967** | -0.09 | 0.92 | 0.09 | -1.00 | 3.15E-01 | -0.14 | 0.87 | 0.08 | -1.86 | 6.23E-02 |
| **GATA3_17662** | -0.12 | 0.89 | 0.08 | -1.51 | 1.31E-01 | -0.16 | 0.85 | 0.07 | -2.28 | 2.25E-02 |
| **GRB7_9249** | 0.03 | 1.03 | 0.20 | 0.15 | 8.83E-01 | -0.04 | 0.96 | 0.20 | -0.19 | 8.51E-01 |
| **GRB7_10859** | 0.05 | 1.05 | 0.18 | 0.29 | 7.73E-01 | 0.04 | 1.04 | 0.17 | 0.23 | 8.19E-01 |
| **GRB7_12564** | -0.02 | 0.98 | 0.10 | -0.20 | 8.38E-01 | -0.05 | 0.95 | 0.09 | -0.51 | 6.12E-01 |
| **ILF2_17207** | 0.24 | 1.27 | 0.20 | 1.21 | 2.27E-01 | 0.22 | 1.24 | 0.18 | 1.18 | 2.39E-01 |
| **LY6D_7343** | 0.20 | 1.23 | 0.10 | 2.02 | 4.31E-02 | 0.26 | 1.30 | 0.09 | 2.96 | 3.12E-03 |
| **LY6D_8723** | 0.16 | 1.18 | 0.08 | 1.97 | 4.86E-02 | 0.19 | 1.21 | 0.07 | 2.66 | 7.85E-03 |
| **LY6D_13503** | 0.27 | 1.31 | 0.12 | 2.24 | 2.52E-02 | 0.28 | 1.32 | 0.11 | 2.60 | 9.31E-03 |
| **MLPH_13960** | -0.09 | 0.91 | 0.13 | -0.71 | 4.78E-01 | -0.20 | 0.82 | 0.12 | -1.72 | 8.46E-02 |
| **MMP7_18414** | -0.05 | 0.95 | 0.08 | -0.62 | 5.34E-01 | -0.01 | 0.99 | 0.07 | -0.11 | 9.15E-01 |
| **MMP7_21090** | -0.07 | 0.93 | 0.08 | -0.87 | 3.83E-01 | -0.01 | 0.99 | 0.08 | -0.15 | 8.84E-01 |
| **MMP9_3124** | -0.03 | 0.97 | 0.18 | -0.16 | 8.72E-01 | 0.05 | 1.05 | 0.16 | 0.33 | 7.43E-01 |
| **MMP9_9922** | -0.05 | 0.95 | 0.12 | -0.45 | 6.54E-01 | -0.05 | 0.95 | 0.11 | -0.42 | 6.75E-01 |
| **MMP9_12495** | -0.07 | 0.93 | 0.11 | -0.65 | 5.18E-01 | -0.03 | 0.97 | 0.10 | -0.31 | 7.58E-01 |
| **MMP9_22262** | -0.09 | 0.91 | 0.16 | -0.57 | 5.68E-01 | 0.01 | 1.01 | 0.14 | 0.07 | 9.44E-01 |
| **MYB_4513** | -0.09 | 0.92 | 0.09 | -1.02 | 3.07E-01 | -0.14 | 0.87 | 0.08 | -1.81 | 7.03E-02 |
| **MYB_5586** | -0.07 | 0.93 | 0.08 | -0.87 | 3.82E-01 | -0.13 | 0.88 | 0.08 | -1.64 | 1.00E-01 |
| **NAT1_9724** | -0.21 | 0.81 | 0.10 | -2.21 | 2.72E-02 | -0.23 | 0.79 | 0.09 | -2.57 | 1.01E-02 |
| **NAT1_13802** | -0.21 | 0.81 | 0.10 | -2.13 | 3.33E-02 | -0.23 | 0.79 | 0.09 | -2.49 | 1.26E-02 |
| **NAT1_18834** | -0.21 | 0.81 | 0.10 | -2.23 | 2.57E-02 | -0.24 | 0.79 | 0.09 | -2.60 | 9.40E-03 |
| **NDRG1_12959** | 0.26 | 1.29 | 0.14 | 1.84 | 6.55E-02 | 0.25 | 1.28 | 0.13 | 1.98 | 4.75E-02 |
| **NDRG1_14278** | 0.27 | 1.31 | 0.14 | 1.90 | 5.80E-02 | 0.27 | 1.31 | 0.13 | 2.07 | 3.83E-02 |
| **OXCT1_13498** | 0.30 | 1.34 | 0.18 | 1.67 | 9.50E-02 | 0.28 | 1.33 | 0.17 | 1.70 | 8.98E-02 |
| **OXCT1_21551** | 0.30 | 1.36 | 0.19 | 1.59 | 1.13E-01 | 0.31 | 1.36 | 0.18 | 1.70 | 8.96E-02 |
| **PERP_10853** | 0.03 | 1.03 | 0.16 | 0.21 | 8.37E-01 | 0.28 | 1.33 | 0.15 | 1.88 | 6.00E-02 |
| **RARA_20979** | -0.54 | 0.58 | 0.49 | -1.12 | 2.64E-01 | -0.88 | 0.41 | 0.44 | -2.00 | 4.54E-02 |
| **RARRES3_5892** | -0.32 | 0.72 | 0.12 | -2.68 | 7.37E-03 | -0.43 | 0.65 | 0.11 | -3.94 | 8.01E-05 |
| **RARRES3_10452** | -0.48 | 0.62 | 0.18 | -2.62 | 8.72E-03 | -0.64 | 0.52 | 0.17 | -3.84 | 1.24E-04 |
| **RARRES3_13636** | -0.53 | 0.59 | 0.18 | -2.89 | 3.80E-03 | -0.71 | 0.49 | 0.17 | -4.28 | 1.89E-05 |
| **SLC39A6_5832** | -0.23 | 0.80 | 0.10 | -2.22 | 2.61E-02 | -0.28 | 0.75 | 0.10 | -2.94 | 3.25E-03 |
| **SLC39A6_10274** | -0.20 | 0.82 | 0.10 | -2.04 | 4.10E-02 | -0.25 | 0.78 | 0.09 | -2.71 | 6.67E-03 |
| **SLC39A6_22525** | -0.23 | 0.79 | 0.10 | -2.33 | 1.98E-02 | -0.28 | 0.75 | 0.10 | -2.96 | 3.08E-03 |
| **SPP1_4283** | 0.05 | 1.05 | 0.11 | 0.46 | 6.47E-01 | 0.14 | 1.15 | 0.10 | 1.37 | 1.71E-01 |
| **SPP1_18222** | 0.04 | 1.04 | 0.10 | 0.35 | 7.26E-01 | 0.14 | 1.15 | 0.10 | 1.42 | 1.55E-01 |
| **TFF1_2352** | -0.03 | 0.97 | 0.07 | -0.41 | 6.82E-01 | -0.07 | 0.93 | 0.07 | -1.03 | 3.03E-01 |
| **TFF1_2679** | 0.01 | 1.01 | 0.08 | 0.13 | 8.99E-01 | -0.05 | 0.95 | 0.08 | -0.63 | 5.30E-01 |
| **TFF1_5677** | -0.01 | 0.99 | 0.07 | -0.20 | 8.45E-01 | -0.05 | 0.95 | 0.07 | -0.80 | 4.26E-01 |
| **TFF1_21524** | -0.04 | 0.96 | 0.07 | -0.57 | 5.70E-01 | -0.08 | 0.93 | 0.06 | -1.18 | 2.36E-01 |
| **TFF3_7479** | 0.23 | 1.26 | 0.23 | 1.02 | 3.08E-01 | 0.04 | 1.04 | 0.22 | 0.20 | 8.45E-01 |
| **TFF3_12400** | -0.08 | 0.92 | 0.06 | -1.48 | 1.39E-01 | -0.12 | 0.88 | 0.05 | -2.33 | 1.99E-02 |
| **XBP1_4265** | -0.32 | 0.72 | 0.10 | -3.28 | 1.05E-03 | -0.42 | 0.66 | 0.09 | -4.68 | 2.90E-06 |
| **XBP1_10024** | -0.38 | 0.68 | 0.10 | -3.72 | 2.01E-04 | -0.46 | 0.63 | 0.09 | -4.88 | 1.04E-06 |
| **XBP1_16753** | -0.30 | 0.74 | 0.10 | -3.02 | 2.52E-03 | -0.40 | 0.67 | 0.09 | -4.47 | 7.94E-06 |
| **MED24_1995** | 0.27 | 1.31 | 0.16 | 1.72 | 8.50E-02 | 0.28 | 1.33 | 0.15 | 1.94 | 5.20E-02 |
| **MED24_8979** | 0.07 | 1.07 | 0.43 | 0.16 | 8.76E-01 | 0.04 | 1.04 | 0.39 | 0.11 | 9.12E-01 |
| **MED24_16803** | 0.16 | 1.18 | 0.16 | 1.03 | 3.04E-01 | 0.17 | 1.18 | 0.15 | 1.13 | 2.57E-01 |
| **MED24_21236** | 0.24 | 1.27 | 0.17 | 1.41 | 1.58E-01 | 0.24 | 1.27 | 0.16 | 1.52 | 1.30E-01 |
